# Low expression of endoplasmic reticulum stress-related gene SERP1 is associated with poor prognosis and immune infiltration in skin cutaneous melanoma

**DOI:** 10.18632/aging.203594

**Published:** 2021-10-05

**Authors:** Yuchao Fan, Xiao Liang, Deshui Yu

**Affiliations:** 1 Department of Anesthesiology, Sichuan Cancer Center, Sichuan Cancer Hospital and Institute, School of Medicine, University of Electronic Science and Technology of China, Chengdu, Sichuan Province, China; 2 Department of Anesthesiology, West China Hospital, Sichuan University, Chengdu, Sichuan Province, China; 3 Department of Anesthesiology, The Second People’s Hospital of Yibin, Yibin, Sichuan Province, China

**Keywords:** endoplasmic reticulum stress, stress-associated endoplasmic reticulum protein 1, skin cutaneous melanoma, prognosis, immune infiltration

## Abstract

Stress-associated endoplasmic reticulum protein 1 (SERP1) is a gene induced by endoplasmic reticulum (ER) stress and a major contributor to multiple tumor types. Skin cutaneous melanoma (SKCM) is a highly aggressive and fatal cancer with poor treatment outcomes after progression. In this study, we evaluated SERP1’s role in tumorigenesis, prognosis, and immune infiltration in SKCM. Patients with SKCM had low SERP1 expression. We identified differentially expressed genes between high- and low-SERP1 expression groups and conducted functional, pathway, and gene enrichment analyses. Protein–protein (PPI) and gene–gene interaction (GGI) networks were constructed via STRING and GeneMANIA, respectively. SERP1 mutation information was obtained through cBioPortal; location in the skin was identified through the Human Protein Atlas. Kaplan–Meier analysis revealed an association between low SERP1 expression and overall survival (OS), disease-specific survival (DSS), progress-free interval (PFI) rates, and worse prognosis in patients with multiple clinicopathological features. Cox regression analysis and nomograms further presented SERP1 level as an independent prognostic factor for patients with SKCM. Furthermore, there were significant correlations between SERP1 expression and immune infiltrates; thus, low SERP1 expression is associated with immune cell infiltration and can be considered a poor prognostic biomarker in patients with SKCM.

Stress-associated endoplasmic reticulum protein 1 (SERP1) is a gene induced by endoplasmic reticulum (ER) stress and a major contributor to multiple tumor types. Skin cutaneous melanoma (SKCM) is a highly aggressive and fatal cancer with poor treatment outcomes after progression. In this study, we evaluated SERP1’s role in tumorigenesis, prognosis, and immune infiltration in SKCM. Patients with SKCM had low SERP1 expression. We identified differentially expressed genes between high- and low-SERP1 expression groups and conducted functional, pathway, and gene enrichment analyses. Protein–protein (PPI) and gene–gene interaction (GGI) networks were constructed via STRING and GeneMANIA, respectively. SERP1 mutation information was obtained through cBioPortal; location in the skin were identified through the Human Protein Atlas. Kaplan–Meier analysis revealed an association between low SERP1 expression and overall survival (OS), disease-specific survival (DSS), progress-free interval (PFI) rates, and worse prognosis in patients with multiple clinicopathological features. Cox regression analysis and nomograms further presented SERP1 level as an independent prognostic factor for patients with SKCM. Furthermore, there were significant correlations between SERP1 expression and immune infiltrates; thus, low SERP1 expression is associated with immune cell infiltration and can be considered a poor prognostic biomarker in patients with SKCM.

## INTRODUCTION

Skin cutaneous melanoma (SKCM) is a common cancer in young adults and the elderly, accounting for 2% of all cancer diagnoses worldwide each year [1]. Melanocytes and pigment-containing cells can transform malignantly into the disease. The incidence of SKCM has steadily globally increased over the past 50 years, with ~96,000 new cases in 2019 [2]. Although SKCM only accounts for about 10% of all skin cancers, it can account for ~80% of all skin cancer deaths [3] and is arguably the most aggressive and lethal type of skin cancer. In 2019, more than 7,000 people died from SKCM in the United States alone [4]. As one of the most difficult solid tumors to treat, managing SKCM is not only a challenge for doctors but also places a heavy financial burden on society [5]. Given that improved survival rates for patients with SKCM can be achieved through early diagnosis [6], it is necessary to find effective biomarkers for diagnosis and prognosis of the disease.

The development and widespread use of next-generation high-throughput sequencing technologies has made available large-scale omics data, such as The Cancer Genome Atlas (TCGA), that allow an in-depth analysis of candidate tumor biomarkers. An increasing number of studies focus on screening genetic biomarkers of SKCM prognosis that are associated with tumor cell invasion, infiltration, and metastasis. Losing CDKN2A, a gene encoding a tumor suppressor protein, is associated with poor prognosis in melanoma patients [7]. Tumor thickness in patients with metastatic melanoma is associated with elevated serum miR-221, which decreases after tumor excision [8]. Additionally, several studies [9, 10] have reported an association between miRNAs and survival of patients with melanoma; therefore, identifying SKCM genetic markers is important for establishing a comprehensive diagnostic and prognostic model.

Stress-associated endoplasmic reticulum protein 1 (SERP1), a Sec61-associated polypeptide induced by endoplasmic reticulum (ER) stress, stabilizes membrane proteins when they are translocating into the lumen of the ER, which prevents unfolded target proteins from degradation during ER stress [11]. Recently, SERP1 has been reported to play an important role in tumor cell survival. Ma et al. [12] reported that SERP1 is a marker of poor prognosis in patients with pancreatic ductal adenocarcinoma. Additionally, high SERP1 levels are associated with poor outcomes in glioblastoma patients [13]; however, studies on the correlations of SERP1 with SKCM prognosis are still lacking. Therefore, this study used large-scale bioinformatics databases to conduct a comprehensive bioinformatics exploration of SERP1 as a prognostic marker for SKCM and investigated the underlying mechanisms.

## RESULTS

### SERP1 expression in pan-cancers and SKCM patients

**Figure 1:**
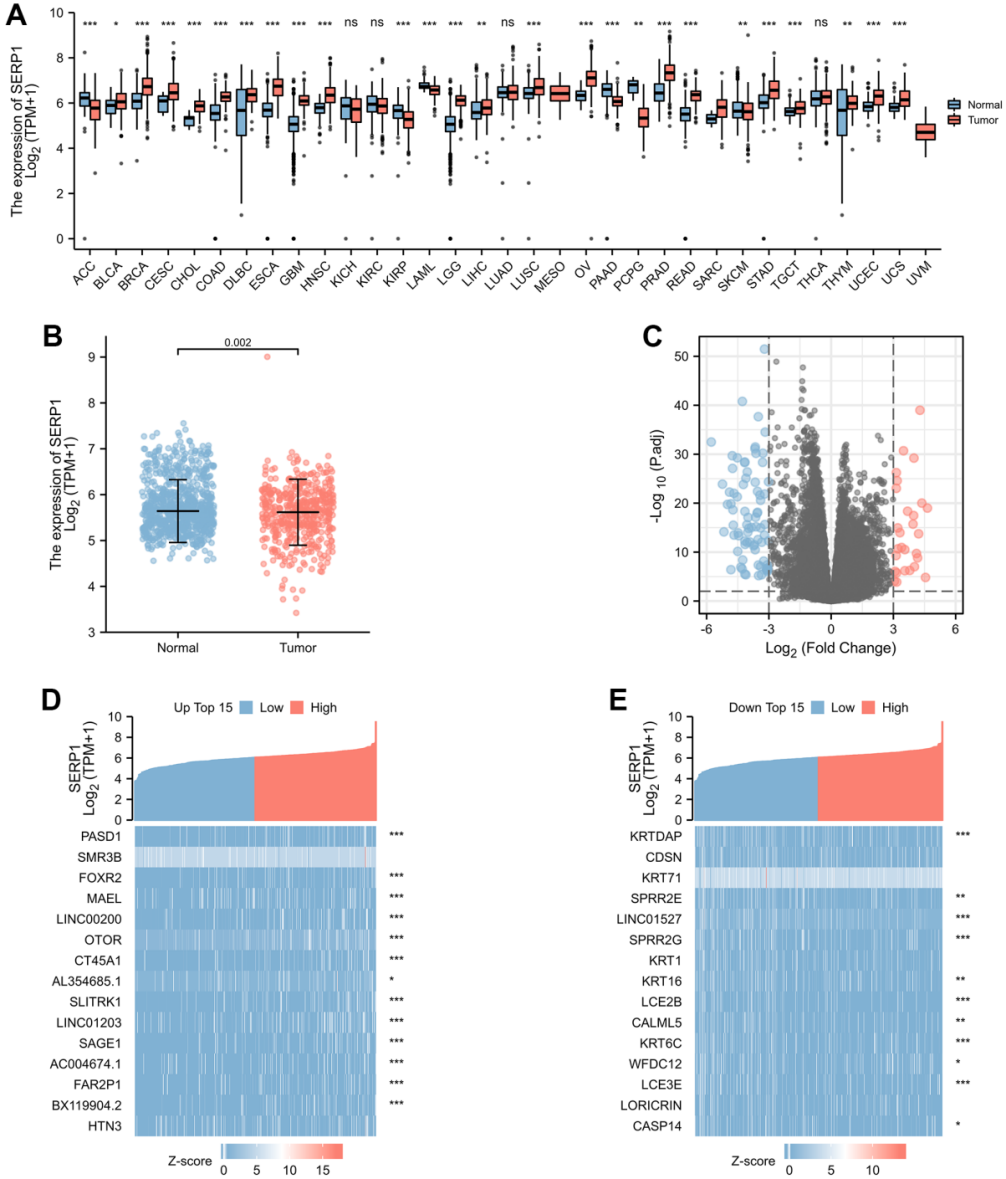
**SERP1 expression in cancers.** (**A**) SERP1 expression in different cancers and normal tissues in TCGA and GTEx pan-cancer data, ns, *p* ≥ 0.05; *p < 0.05; **p < 0.01; ***p < 0.001, (**B**) The SERP1 expression in SKCM and normal tissues, (**C**) The volcano plots of DEGs between high and low SERP1 expression groups, (**D**) The heatmap of top 15 up-regulated DEGs, (**E**) The heatmap of top 15 down-regulated DEGs.

**Figure 2:**
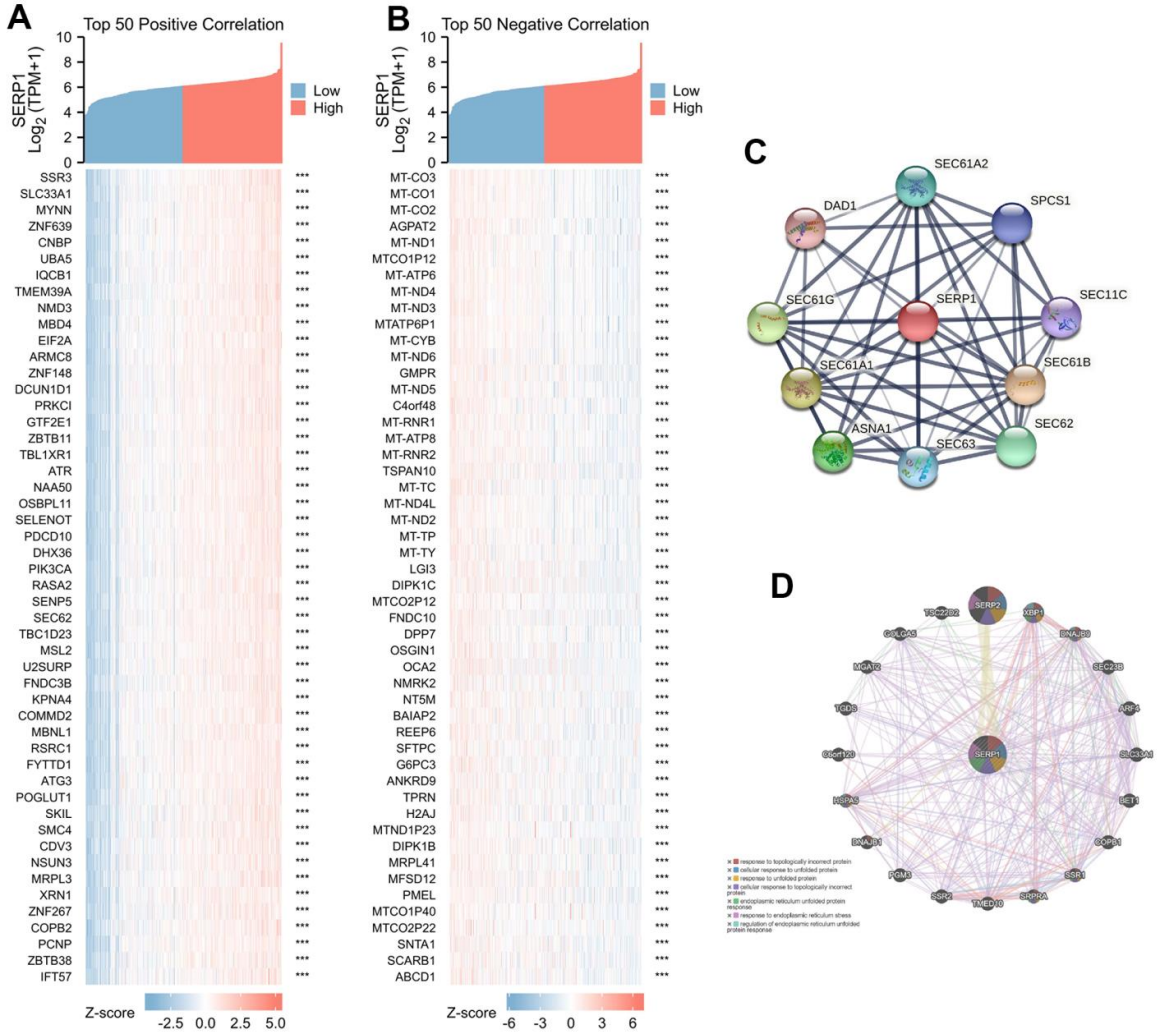
**Genes related to SERP1.** (**A**) The heatmap of top 50 positively RNAs related to SERP1, (**B**) The heatmap of top 50 negative RNAs related to SERP1, (**C**) The PPI network of SERP1 built via STRING, (**D**) The GGI network of SERP1 built via GeneMANIA.

The association between SERP1 expression and clinical characteristics in SKCM patients were list in Table 1. By analyzing The Cancer Genome Atlas (TCGA) and Genotype-Tissue Expression (GTEx) database, we obtained the expression of SERP1 RNA in pan-cancer. As Figure 1A revealed, compared to normal tissues, there was significantly different expression of SERP1 mRNA in 33 incorporated cancers except KICH, KIRC and THCA. Because there were only tumor-related samples without normal tissue samples (MESO and UVM) or too few normal tissue samples (SARC having 2 samples) in the database for these three cancer types, these three cancers could not be compared to normal tissue in the pan-cancer comparison and therefore no results are shown. As the target of our study, SERP1 expression was lower in SKCM tumors than in normal tissues (p = 0.002, Figure 1B). Then SKCM patients were divided into the high SERP1 expression group and low SERP1 expression group based on the median SERP1 expression. We compared the RNA expression between these two groups. There were 111 RNAs that met the established selected threshold and were recognized as differentially expressed genes (DEGs) (Adjust P-value < 0.05 and absolute log-fold change > 3) (Figure 1C), of which 30 were upregulated (logFC is positive) and 81 were downregulated (logFC is negative). Top 15 up-regulated and down-regulated DEGs were illustrated by heatmaps and included in the table (Figure 1D, 1E and Supplementary Table 2). We also analyzed RNAs correlated with SERP1 expression in SKCM patients and showed the top 50 positively and negatively associated RNAs as heatmaps (Figure 2A, 2B and Supplementary Table 3).

**Table 1:** The association between SERP1 expression and clinical characteristics in SKCM patients.

**Characteristic**	**Low expression of SERP1**	**High expression of SERP1**	**p**	**Method**
n	235	236		
Gender, n (%)			0.138	Chisq.test
Female	81 (17.2%)	98 (20.8%)		
Male	154 (32.7%)	138 (29.3%)		
Race, n (%)			0.771	Fisher.test
Asian	7 (1.5%)	5 (1.1%)		
Black or African American	0 (0%)	1 (0.2%)		
White	224 (48.6%)	224 (48.6%)		
Age, n (%)			0.056	Chisq.test
<=60	115 (24.8%)	137 (29.6%)		
>60	116 (25.1%)	95 (20.5%)		
Weight, n (%)			0.284	Chisq.test
<=70	36 (13.9%)	41 (15.8%)		
>70	100 (38.6%)	82 (31.7%)		
Height, n (%)			0.753	Chisq.test
< 170	64 (25.2%)	54 (21.3%)		
>=170	70 (27.6%)	66 (26%)		
BMI, n (%)			0.590	Chisq.test
<=25	42 (16.7%)	42 (16.7%)		
>25	91 (36.3%)	76 (30.3%)		
T stage, n (%)			0.004	Chisq.test
T1	17 (4.7%)	24 (6.6%)		
T2	34 (9.3%)	45 (12.4%)		
T3	45 (12.4%)	46 (12.6%)		
T4	98 (26.9%)	55 (15.1%)		
N stage, n (%)			0.192	Chisq.test
N0	126 (30.4%)	109 (26.3%)		
N1	31 (7.5%)	43 (10.4%)		
N2	28 (6.8%)	21 (5.1%)		
N3	25 (6%)	31 (7.5%)		
M stage, n (%)			0.076	Chisq.test
M0	219 (49.4%)	199 (44.9%)		
M1	8 (1.8%)	17 (3.8%)		
Pathologic stage, n (%)			< 0.001	Chisq.test
Stage I	32 (7.8%)	45 (10.9%)		
Stage II	90 (21.8%)	50 (12.1%)		
Stage III	79 (19.2%)	92 (22.3%)		
Stage IV	8 (1.9%)	16 (3.9%)		
Radiation therapy, n (%)			0.031	Chisq.test
No	200 (43.1%)	183 (39.4%)		
Yes	31 (6.7%)	50 (10.8%)		
Tumor tissue site, n (%)			0.658	Chisq.test
Extremities	96 (22.9%)	101 (24.1%)		
Trunk	92 (22%)	79 (18.9%)		
Head and Neck	22 (5.3%)	16 (3.8%)		
Other Specify	7 (1.7%)	6 (1.4%)		
Melanoma ulceration, n (%)			0.361	Chisq.test
No	76 (24.2%)	71 (22.6%)		
Yes	96 (30.6%)	71 (22.6%)		
Melanoma Clark level, n (%)			0.010	Fisher.test
I	6 (1.9%)	0 (0%)		
II	9 (2.8%)	9 (2.8%)		
III	30 (9.3%)	47 (14.6%)		
IV	94 (29.2%)	74 (23%)		
V	32 (9.9%)	21 (6.5%)		
Breslow depth, n (%)			0.002	Chisq.test
<=3	85 (23.6%)	100 (27.8%)		
>3	110 (30.6%)	65 (18.1%)		
Age, median (IQR)	61 (51, 72)	56 (45.75, 69.25)	0.003	Wilcoxon

Abbreviations: SERP1, Stress associated endoplasmic reticulum protein 1; SKCM, Skin Cutaneous Melanoma; IQR, interquartile range.

### Protein-protein interaction (PPI) and gene–gene interaction (GGI) network analyses of SERP1 in SKCM patients

The PPI network analysis was conducted to explore the potential interactions of SERP1 protein. As Figure 2C presents, the network with interaction nodes and edges was built via STRING, whose top 10 proteins are listed in Supplementary Table 4. The GGI network also showed that the functions of the differentially expressed SERP1 and its associated genes (such as SERP2, XBP1, DNAJB9, SEC23B, ARF4, SLC33A1, BET1, COPB1, SSR1, SRPRA, TMED10, SSR2, PGM3, DNAJB1, HSPA5, C6orf120, TGDS, MGAT2, GOLGA5, TSC22D2) were primarily related to response to topologically incorrect protein, cellular response to unfolded protein, response to unfolded protein, cellular response to topologically incorrect protein, endoplasmic reticulum unfolded protein response, response to endoplasmic reticulum stress and regulation of endoplasmic reticulum unfolded protein response (Figure 2D).

### Predicted functions and pathways of the DEGs between high- and low-SERP1 expression in SKCM patients

**Figure 3:**
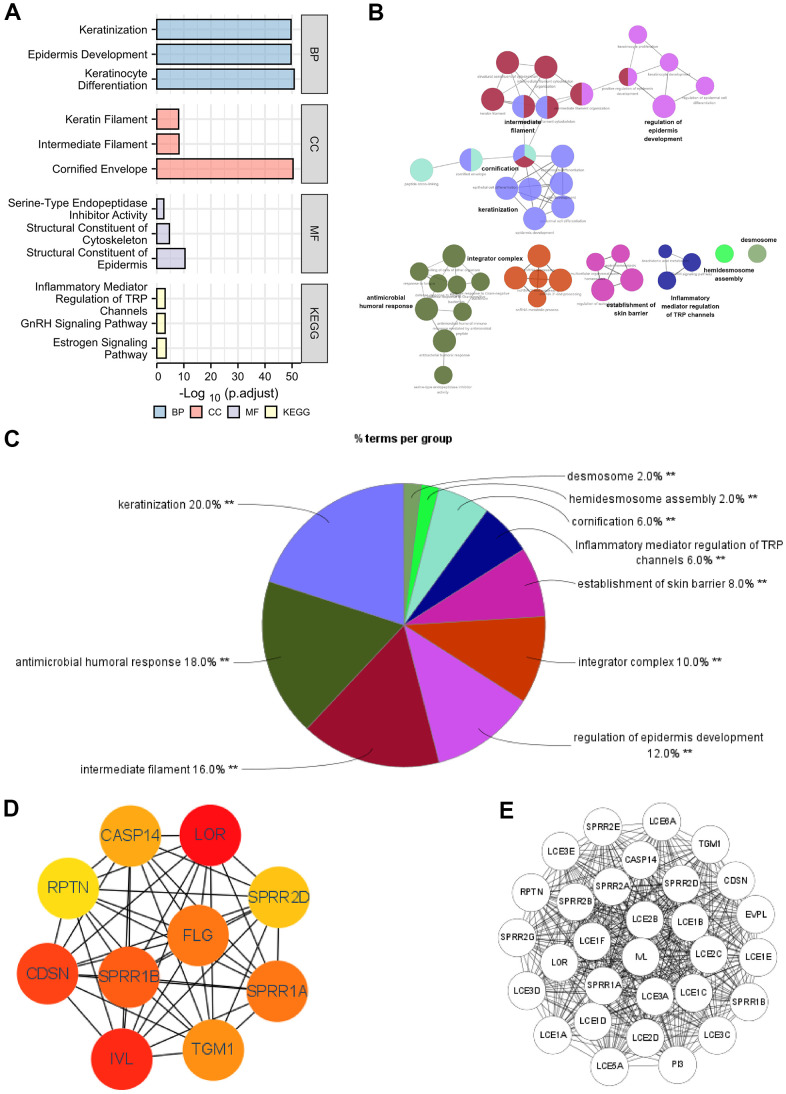
**Functional enrichment analysis of DEGs between high and low expression of SERP1 in SKCM patients.** (**A**–**C**) GO and KEGG pathway enrichment analyses for DEGs between High and -Low expression of SERP1 in SKCM patients. (**D**) The top 10 hub genes ranked by MCC of cytoHubba, (**E**) The top 30 hub genes ranked by MCODE.

The functions of the DEGs between high- and low- SERP1 expression groups were predicted by analyzing Gene Ontology (GO) function and Kyoto Encyclopedia of Genes and Genomes (KEGG) via R software and Cytoscape software. The different RNA functional of DEGs included three categories: the biological process (BP), molecular function (MF), and cellular component (CC). The top three GO terms of BP, CC and MF functional groups found via R software are present as Figure 3A.

The BP ontology included keratinization, epidermis development and keratinocyte differentiation. In the CC ontology, most genes were connected with cornified envelope, intermediate filament and keratin filament. In addition, the structural constituent of epidermis, structural constituent of cytoskeleton with serine-type endopeptidase inhibitor activity made up the majority of MF ontology. Furthermore, the KEGG pathway analysis showed that the DEGs were most closely correlated to Estrogen signaling pathway, GnRH signaling pathway and Inflammatory mediator regulation of TRP channels. Detailed results of GO and KEGG are placed in Supplementary Table 5.

On the other hand, we also performed GO and KEGG analysis of DEGs using ClueGO and the results were presets as Figure 3B. Results followed cut-off threshold P-value < 0.01 were just enriched in BP, CC and KEGG (Figure 3C). The BP ontology included keratinization (20%), antimicrobial humoral response (18%), regulation of epidermis development (12%), establishment of skin barrier (8%), cornification (6%) and hemidesmosome assembly (2%). The CC ontology included integrator complex (10%), intermediate filament (16%), and desmosome (2%). The KEGG pathway was Inflammatory mediator regulation of TRP channels (6%). The top 10 nodes ranked by MCC of cytoHubba were SPRR1B, CDSN, RPTN, IVL, SPRR2G, LOR, SPRR2E, EVPL, PI3 and TGM1 (Figure 3D) and modules with MCODE score = 30 including LCE1C, SPRR2A, LCE3E, LCE3D, LCE2D, LCE2C, LCE1E, SPRR1A, SPRR2D, SPRR2B, SPRR2E, SPRR2G, LCE1B, LCE3A, LCE1A, LCE3C, LCE5A, RPTN, LCE1D, SPRR1B, PI3, LOR, IVL, CASP14, LCE6A, CDSN, TGM1, LCE2B, LCE1F, EVPL were present in Figure 3E.

### Gene set enrichment analyses (GSEA) identifies DEGs between high- and low-SERP1 expression related signaling pathways

**Figure 4:**
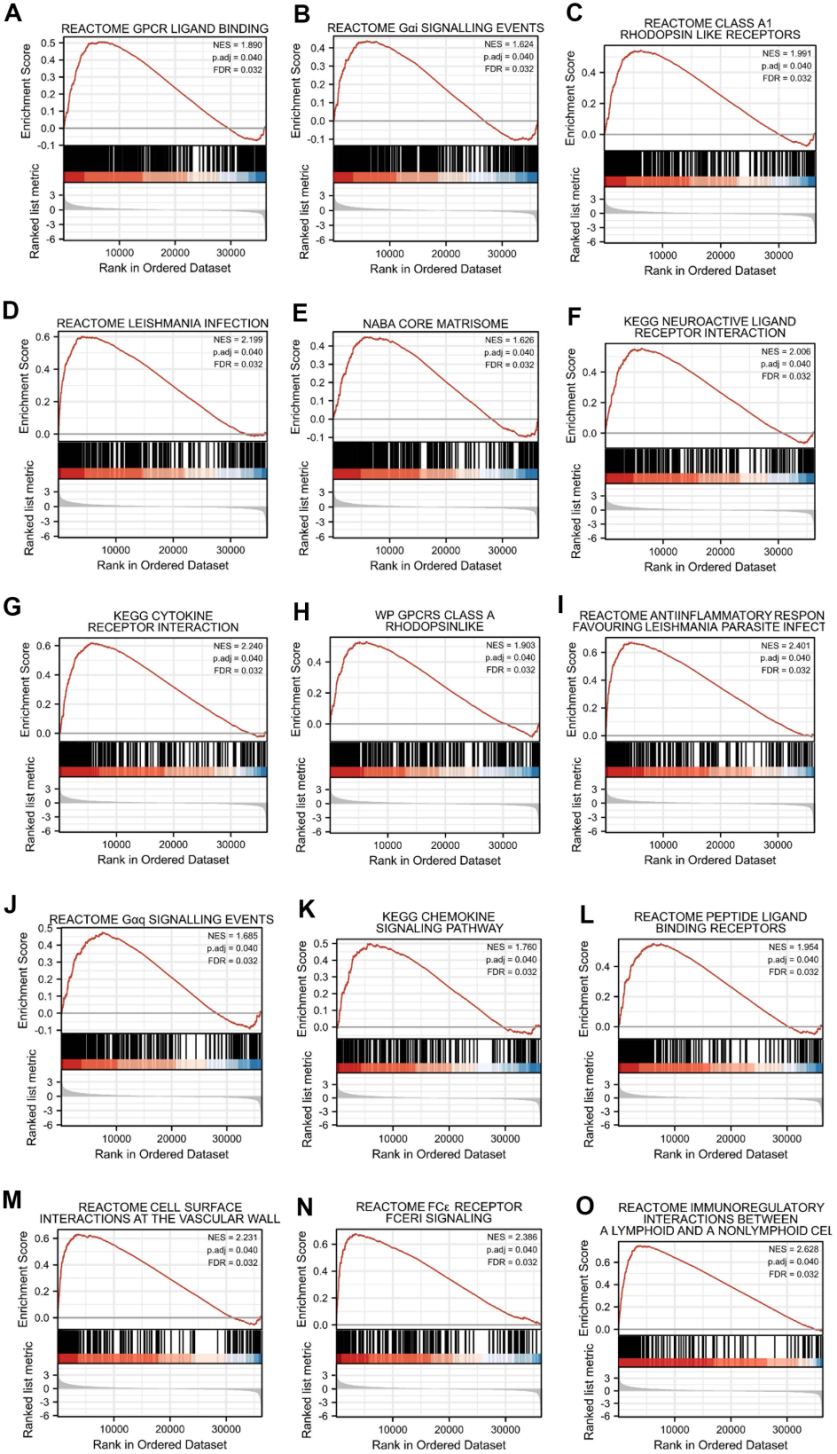
**Top 15 enrichment plots of GSEA.** The GSEA results showed that DEGs involved in (**A**) Reactome GPCR ligand binding, (**B**) Reactome G alpha i signalling events, (**C**) Reactome class a1 rhodopsin like receptors, (**D**) Reactome leishmania infection, (**E**) Naba core matrisome, (**F**) Kegg neuroactive ligand receptor interaction, (**G**) Kegg cytokine receptor interaction, (**H**) Wp GPCRs class a rhodopsin-like, (**I**) Reactome anti-inflammatory response favouring leishmania parasite infection, (**J**) Reactome G alpha q signalling events, (**K**) Kegg chemokine signaling pathway, (**L**) Reactome peptide ligand binding receptors, (**M**) Reactome cell surface interactions at the vascular wall, (**N**) Reactome FC epsilon receptor (FCERI) signaling and (**O**) Reactome immunoregulatory interactions between a lymphoid and a nonlymphoid cell.

We additionally conducted GSEA to identify signaling pathways that were differentially activated between high- and low-SERP1 expression groups in SKCM. The results indicated the Top 15 pathways were associated with Reactome GPCR ligand binding, Reactome G alpha i signaling events, Reactome class a1 rhodopsin like receptors, Reactome leishmania infection, Naba core matrisome, Kegg neuroactive ligand receptor interaction, Kegg cytokine-cytokine receptor interaction, Wp GPCRs class a rhodopsin-like, Reactome anti-inflammatory response favouring leishmania parasite infection, Reactome G alpha q signaling events, Kegg chemokine signaling pathway, Reactome peptide ligand binding receptors, Reactome cell surface interactions at the vascular wall, Reactome FC epsilon receptor (FCERI) signaling and Reactome immunoregulatory interactions between a lymphoid and a nonlymphoid cell (Figure 4A–4O). We further list the Top 50 pathways in Supplementary Table 6.

### Genetic alteration and protein localization of SERP1 in SKCM patients

**Figure 5:**
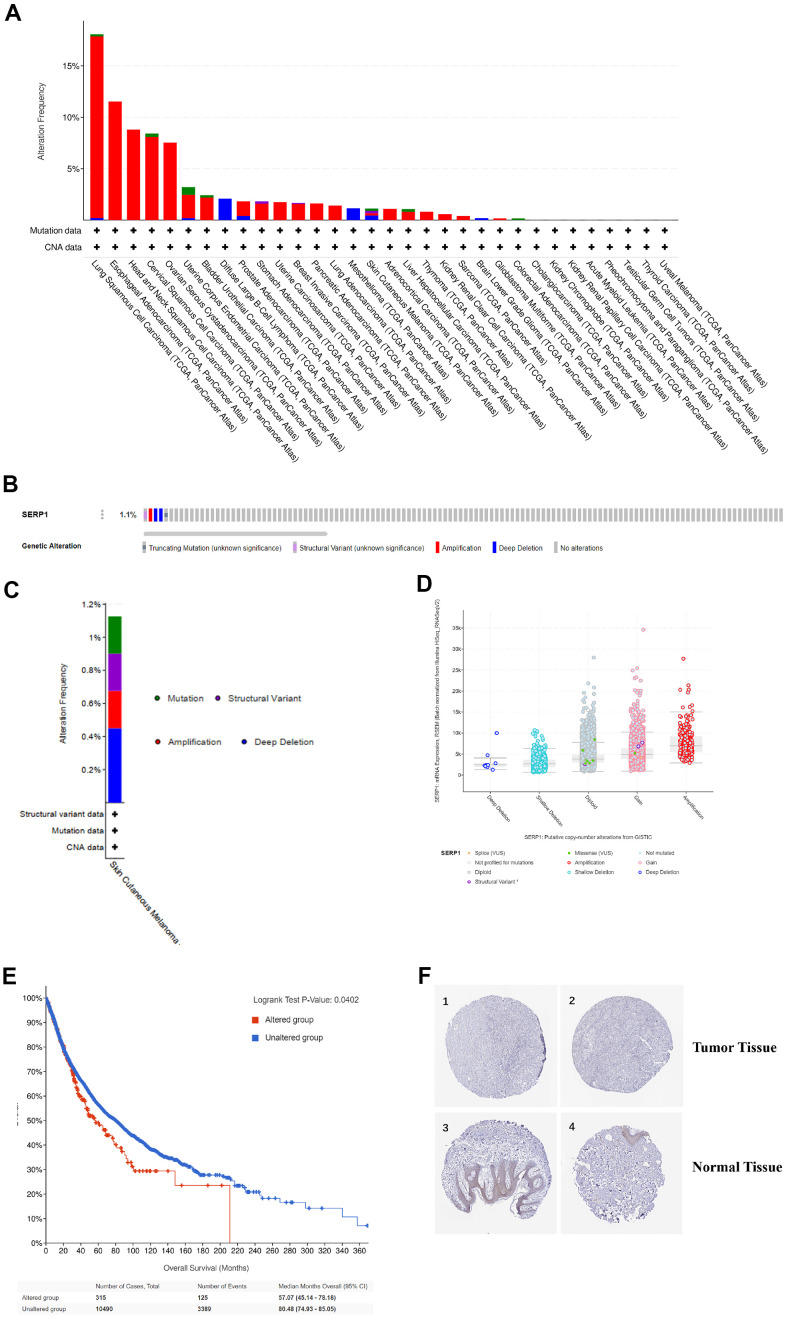
**Genetic alteration and protein localization of SERP1 in SKCM patients.** (**A**) Bar chart of SERP1 mutation in pan-cancers based on TCGA database. (**B**) SERP1 gene expression and mutation analysis in SKCM. (**C**) The distribution of SERP1 genomic alterations in SKCM. (**D**) The graph of the correlation between SERP1 expression and copy number alterations in SKCM. (**E**) Kaplan-Meier curve of OS in SKCM patients with altered (red) and unaltered (blue) mRNA expression of the SERP1 gene. (**F**) The representative IHC staining images from HPA database presents SERP1 expression in normal and tumor tissues.

We analyzed the genetic mutations of SERP1 expression in SKCM patients via the cBioPortal online tool. Based on TCGA, SERP1 mutated lowly in SKCM via pan-cancers analysis (Figure 5A). Figure 5B showed the mutation rate of SERP1 genes was 1.1%. Figure 5C indicated the major form alterations of the SERP1 genes in SKCM is amplification. Figure 5D presented there was an overall higher amount of amplification occurrence of SERP1 mRNA expression in SKCM patients. We further explored the correlation between genetic mutations and the prognosis of SKCM patients. The statistically significant result was showed in Figure 5E that SERP1 genetic mutations decrease overall survival (OS) of SKCM patients (p = 0.0402). However, it is noteworthy that the expression of SERP1 protein did not show significant differences between tumor tissues and normal tissues in the Human Protein Atlas (Figure 5F).

### Correlations between SERP1 and prognosis in SKCM patients

**Figure 6:**
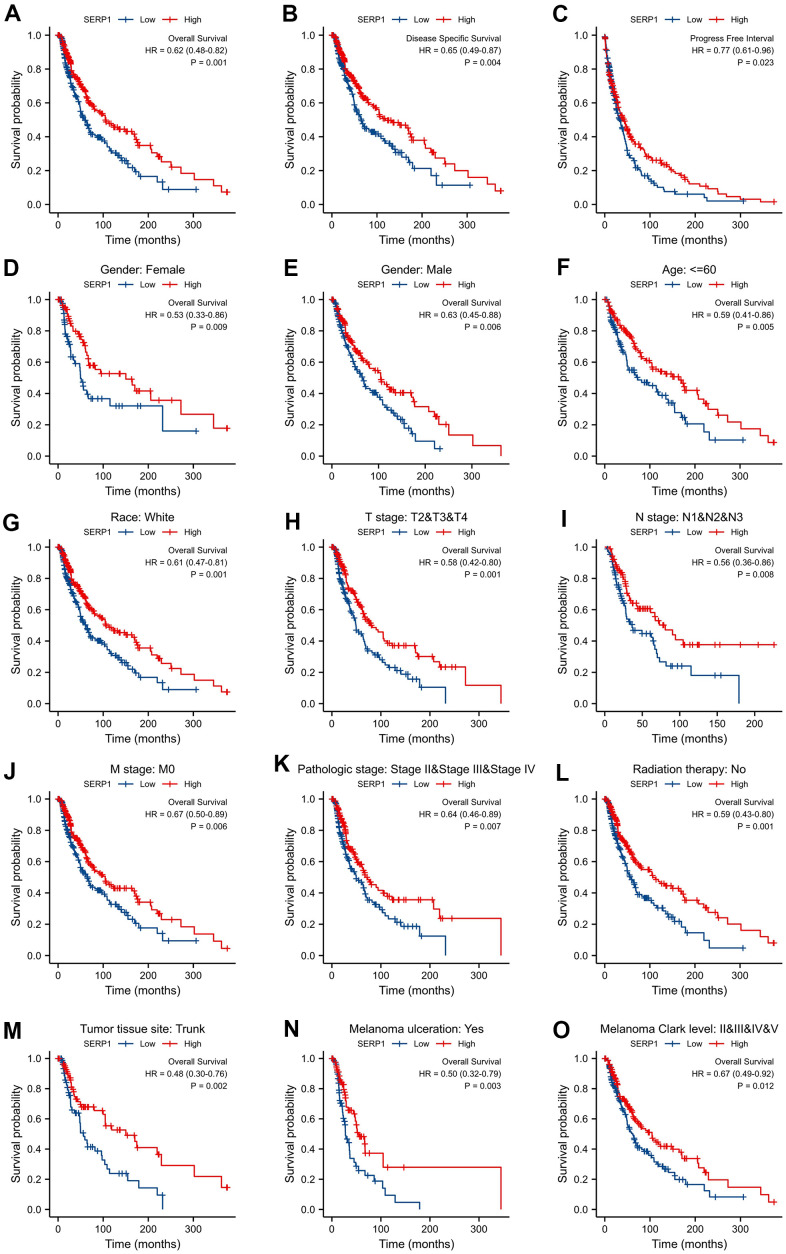
**Correlations between SERP1 and prognosis in SKCM patients.** (**A**) OS Kaplan-Meier curve for SERP1 in SKCM patients, (**B**) DSS Kaplan-Meier curve, (**C**) PFI survival Kaplan-Meier curve, (**D**–**O**) OS Kaplan-Meier curve of statistically significant subgroups for (**D**) Female, (**E**) male, (**F**) Age≤60, (**G**) Race white, (**H**) T stage (T2-T4), (**I**) N stage (N1-N3), (**J**) M stage (M0), (**K**) Pathologic Stage (Stage II-IV), (**L**) Radiation therapy No, (**M**) Tumor tissue site Trunk, (**N**) Melanoma ulceration Yes, (**O**) Melanoma Clark Level (Stage II-V).

In order to explore the prognosis value of SERP1 in SKCM patients, we performed Kaplan-Meier analysis to evaluate its value on prediction of SKCM on clinical outcomes. The result found that OS (hazard ratio (HR): 0.62, 95% confidence interval (CI): 0.48–0.82, p = 0.001 (Figure 6A), disease specific survival (DSS) (HR: 0.65, 95% CI 0.49-0.87, p = 0.004) (Figure 6B) and progress free interval (PFI) (HR: 0.77, 95% CI 0.61-0.96, p = 0.023) (Figure 6C) for high SERP1 groups were all statistically better than those for the low SERP1 groups. In addition, in order to comprehensive understand the multidimensional prospect of the correlation of SERP1 expression to SKCM patient’s survival, subgroup Kaplan-Meier analysis of OS was also performed according to different clinical variables. As Figure 6D–6O revealed, low SERP1 expression was significantly associated with worse OS in SKCM patients both of gender, female (p = 0.009) or male (p = 0.006), race white (p = 0.001), age ≤ 60 (p = 0.005), T stage is T2-4 (p = 0.001), N stage is N1-3 (p = 0.008), M stage is 0 (p = 0.006), Pathologic Stage is II-IV (p = 0.007), without radiation therapy (p = 0.001), Tumor tissue site is on the trunk (p = 0.003), having melanoma ulceration (p = 0.003) and Melanoma Clark Level Stage is II-V (p = 0.012). However, the high or low SERP1 expression on other cases did not show statistical significance on OS (Supplementary Figure 1A–1L).

**Figure 7:**
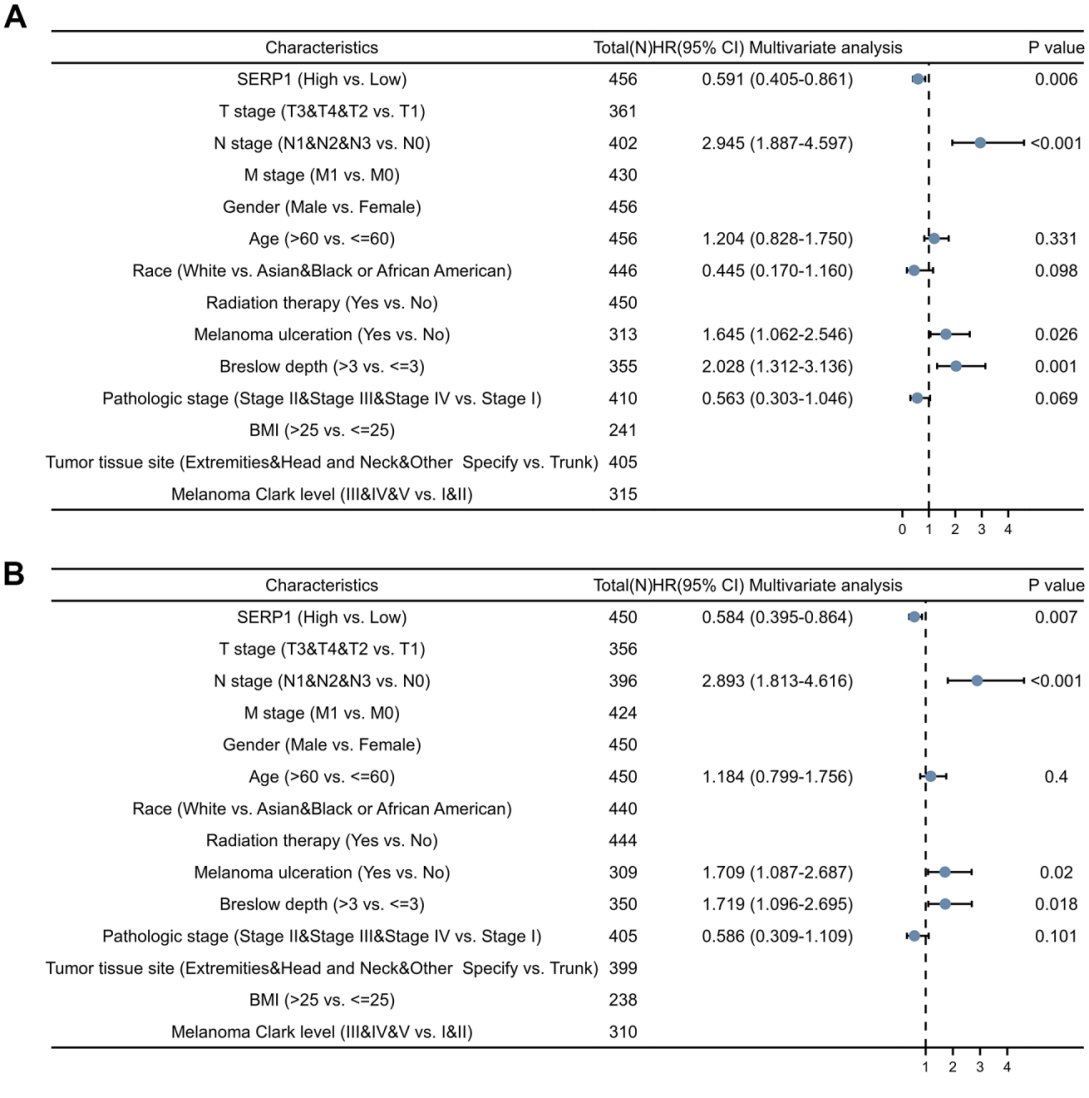
**Forest plots of different clinical variables for SERP1 in SKCM patients.** Forest plot of different clinical variables on OS (**A**) and DSS (**B**) by multivariate cox regression analysis.

Univariate Cox analysis showed that SERP1 expression was an independent risk factor for OS (HR: 0.591, 95% CI 0.405-0.861, p = 0.006) (Figure 7A and Supplementary Table 7) and DSS (HR: 0.584, 95% CI 0.395-0.864, p = 0.007) (Figure 7B and Supplementary Table 8). However, SERP1 expression did not have significant predictive power for PFI (HR: 0.769, 95% CI 0.555-1.067, p = 0.116) (Supplementary Table 9). Also available as independent risk factors through Univariate Cox analysis were N stage (HR: 2.945, 95% CI 1.887-4.597, p < 0.001), Melanoma ulceration (HR: 1.645, 95% CI 1.062-2.546, p = 0.026) and Breslow depth (HR: 2.028, 95% CI 1.312-3.136, p = 0.001) for OS and N stage (HR: 2.893, 95% CI 1.813-4.616, p < 0.001), Melanoma ulceration (HR: 1.709, 95% CI 1.087-2.687, p = 0.020) and Breslow depth (HR: 1.719, 95% CI 1.096-2.695, p = 0.018) for DSS, respectively.

**Figure 8:**
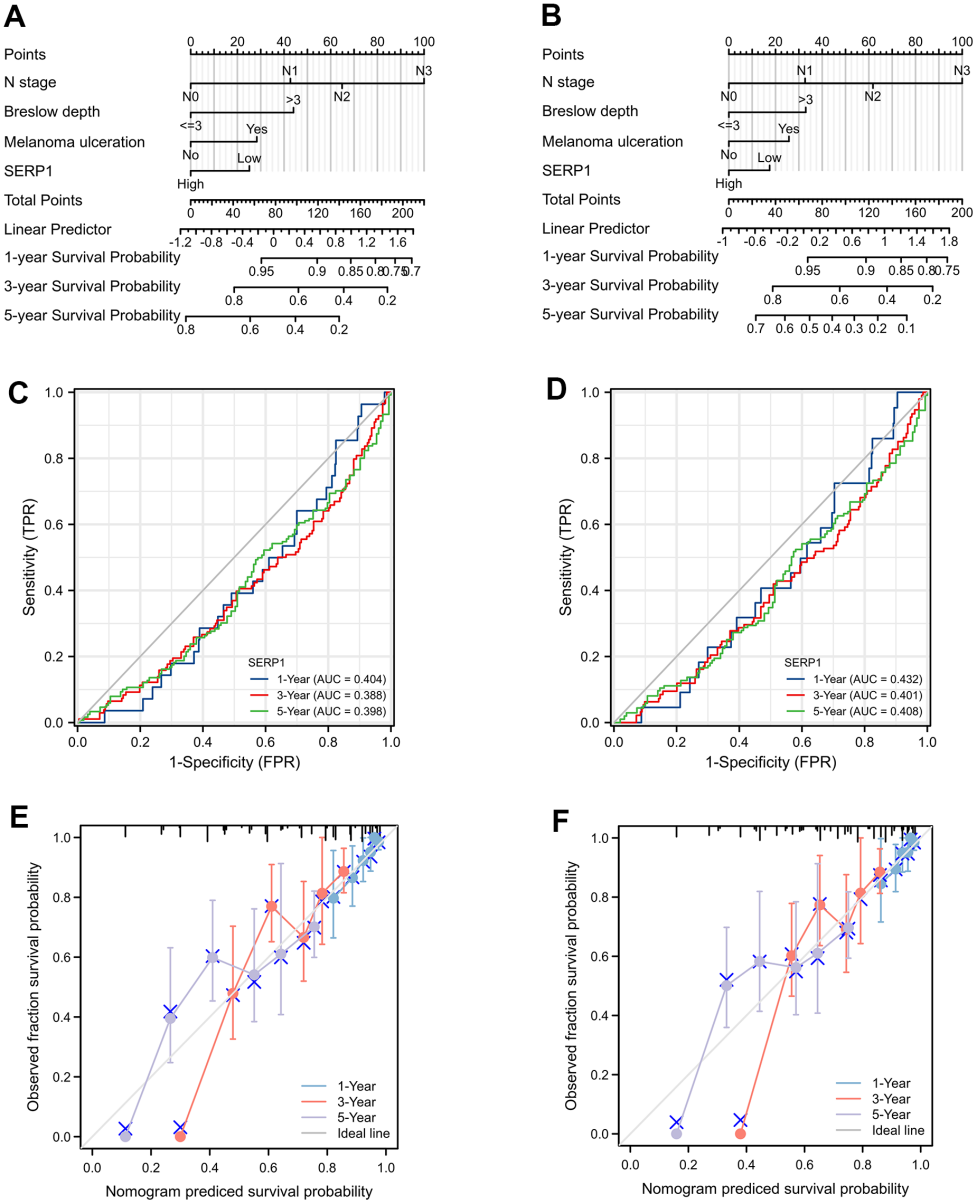
**The prognostic nomogram for predicting OS and DSS probability.** (**A**) The prognostic nomogram for predicting OS probability by the multivariable Cox regression model via the four statistically significant predictors, such as SERP1, N stage, Melanoma ulceration and Breslow depth. (**B**) The prognostic nomogram for predicting DSS probability. (**C**) The time-dependent ROC curves of OS for 1, 3, 5 year. (**D**) The time-dependent ROC curves of DSS for 1, 3, 5 year. (**E**) The calibration curve of OS for 1, 3, 5 year. (**F**) The calibration curve of DSS for 1, 3, 5 year.

The nomograms predicting OS (Figure 8A) and DSS (Figure 8B) was constructed based on significant risk factors identified from in the univariate Cox analyses. The score of the corresponding risk factors should be first identified by the point scale at the top of the nomogram. All scores were then summed and the corresponding 1-year, 3-year, and 5-year survival probability was obtained corresponding to the scales at the bottom of the nomogram. The time-dependent ROC curves of OS and DSS are shown in Figure 8C, 8D, respectively. The 1, 3, and 5-year AUCs of OS are 0.404 (95% CI 0.308-0.500), 0.388 (95% CI 0.324-0.453) and 0.398 (95% CI 0.334-0.463), respectively. The 1, 3, and 5-year AUCs of DSS are 0.432 (95% CI 0.324-0.539), 0.401 (95% CI 0.333-0.469) and 0.408 (95% CI 0.341-0.475), respectively. These suggesting that low SERP1 has a good predictive efficacy and diagnostic accuracy for survival of SKCM patients. Details of the ROC information are presented in Supplementary Table 10.

Calibration curves showed a strong consistency between the possibilities obtained by the nomogram and the real results of 1-year and good consistency of 3 and 5-year for OS (Figure 8E) and DSS (Figure 8F). Furthermore, the C-index values were 0.686 (95% CI 0.662 to 0.710) and 0.664 (95% CI 0.638 to 0.690) for OS and DSS, respectively. These revealed the good credibility of the nomogram.

### Correlation between SERP1 expression and clinical variables in SKCM patients

**Figure 9:**
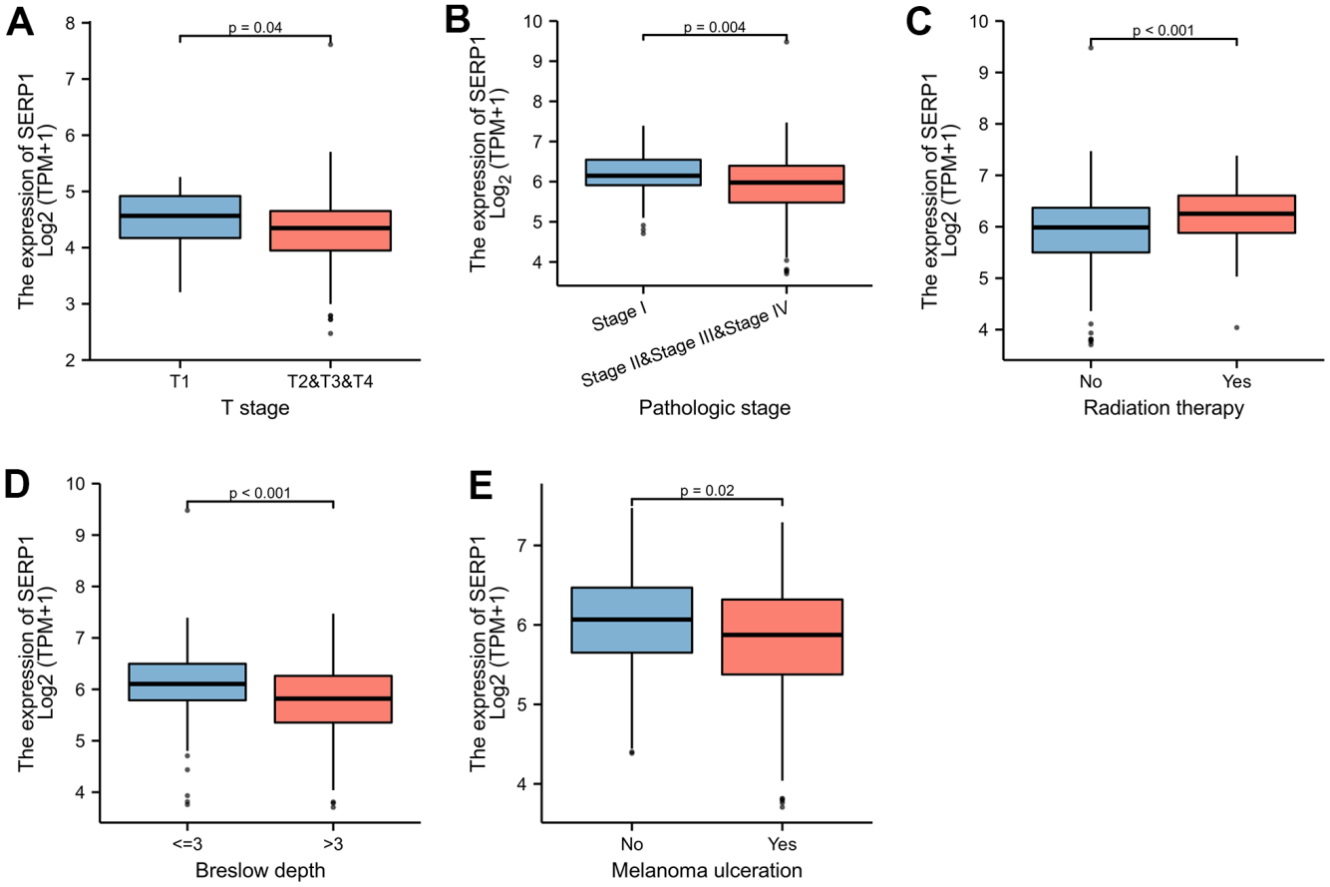
**SERP1 expression is associated with different clinical variables in SKCM patients.** (**A**) T classification, (**B**) Pathologic stage, (**C**) Radiation therapy, (**D**) Breslow depth, (**E**) Melanoma ulceration.

The Figure 9A–9E revealed the correlation between the expression of SERP1 and the different clinical variables of SKCM patients. SERP1 expression level significantly related to the T stage (p = 0.037), Pathologic stage (p = 0.009), Radiation therapy (p = 0.013), Breslow depth (p < 0.001) and Melanoma ulceration (p = 0.043). The higher SERP1 expression was associated with lower T stage and Pathologic stage, shallower Breslow depth and fewer ulceration. Moreover, the expression of SERP1 was lower in patients with radiation therapy experience.

### Correlation of SERP1 and immune cell infiltration in SKCM

**Figure 10:**
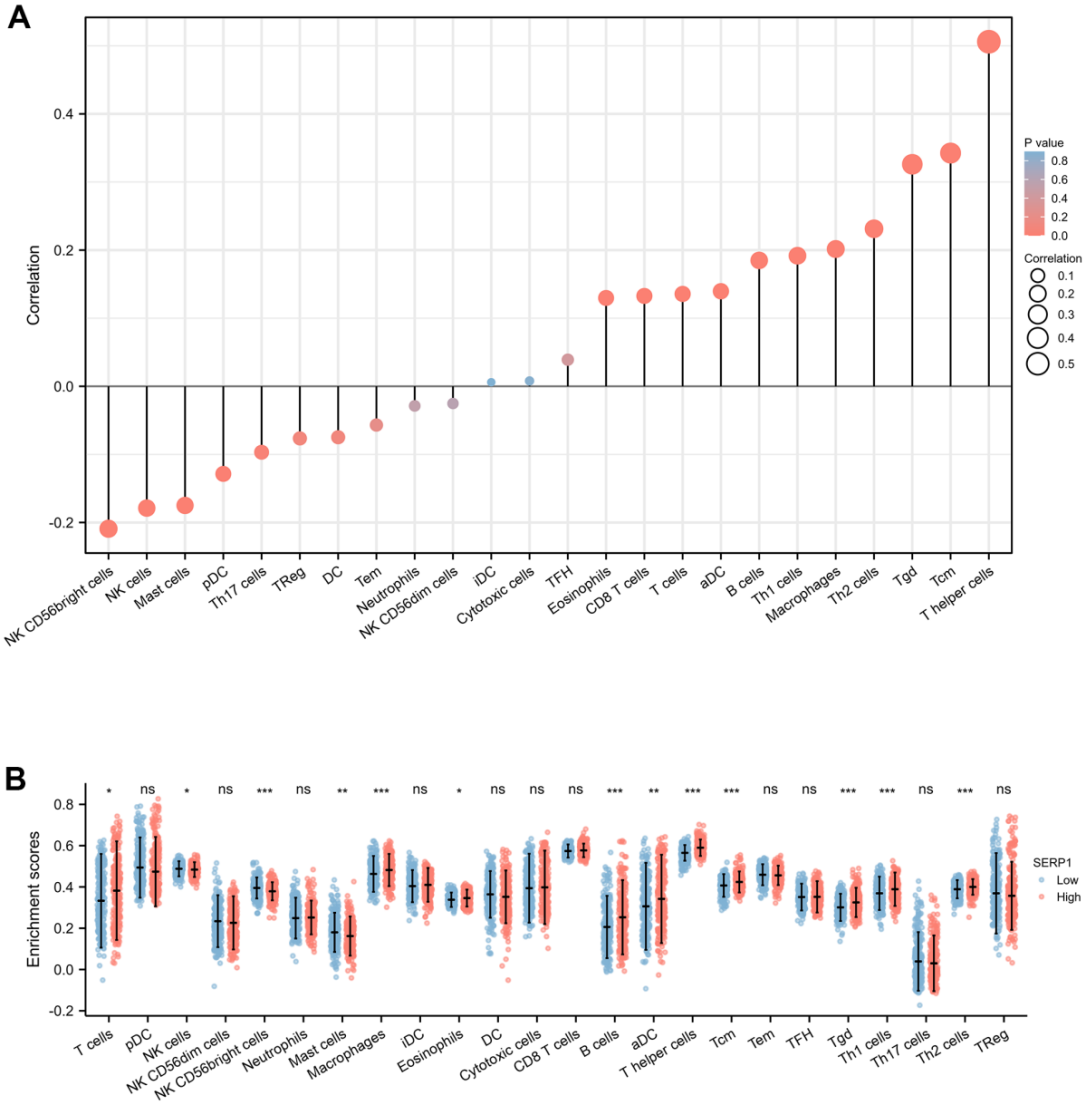
**Associations of SERP1 expression and immune infiltration level in SKCM patients.** (**A**) Correlation of SERP1 expression with immune infiltration level of 24 immune cell types by Spearman’s analysis. (**B**) Twenty-four types of immune cells are plotted according to different SERP1 expression levels.* *p*<0.05, ** *p*<0.01, *** *p*<0.001.

**Figure 11:**
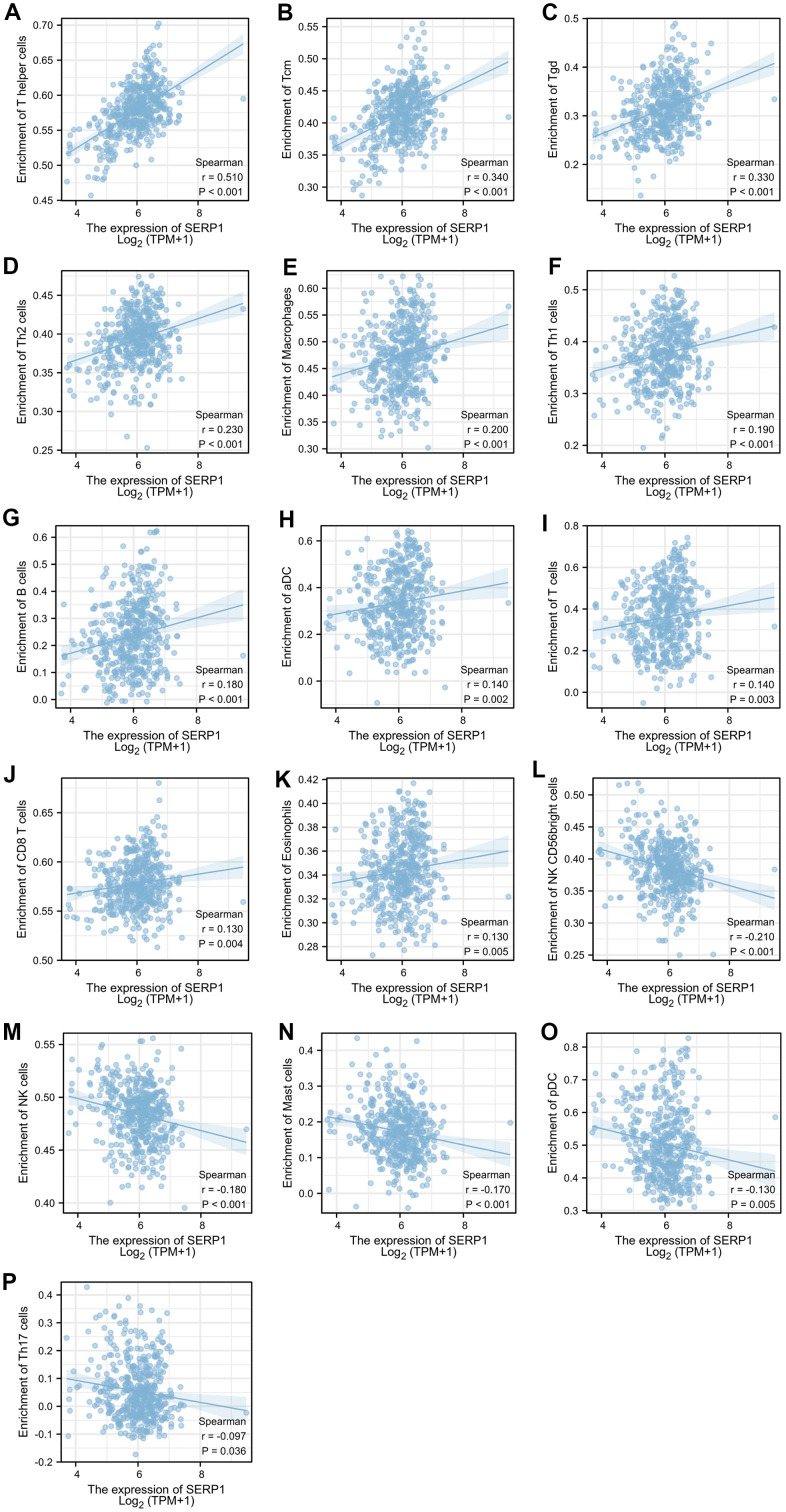
**Relationship of SERP1 expression with immune cell level in SKCM.** (**A**–**P**) SERP1 expression showed significant positive related to infiltrating levels of T helper cells, Tcm, Tgd, Th2 cells, Macrophages, Th1 cells, B cells, aDC, T cells, CD8 T cells, Eosinophils and significant negative related to infiltrating levels of NK CD56 bright cells, NK cells, Mast cells, pDC and Th17 cells.

The results found that T helper cells (r = 0.510, p < 0.001), Tcm (r = 0.340, p < 0.001), Tgd (r = 0.330, p < 0.001), Th2 cells (r = 0.230, p < 0.001), Macrophages (r = 0.200, p < 0.001), Th1 cells (r = 0.190, p < 0.001), B cells (r = 0.180, p < 0.001), aDC (r = 0.140, p = 0.002), T cells (r = 0.140, p = 0.003), CD8 T cells (r = 0.130, p = 0.004), Eosinophils (r = 0.130, p = 0.005) showed positive association with SERP1 expression. The NK CD56 bright cells (r = -0.210, p < 0.001), NK cells (r = -0.180, p < 0.001), Mast cells (r = -0.170, p < 0.001), pDC (r = -0.130, p = 0.005), Th17 (r = -0.097, p = 0.036) were negatively correlated with SERP1 (Figures 10A, 11). However, the TFH, Cytotoxic cells, iDC, NK CD56dim cells, Neutrophils, Tem, DC, Treg showed no significant correlation with SERP1 (Supplementary Figure 2).

We further evaluated the infiltration levels of 24 immune cells above in high- or low-SERP1 expression group (Figure 10B). The result indicated in high SERP1 expression group, the T cells (p = 0.018), aDC (p = 0.009), B cells (p < 0.001), Eosinophils (p = 0.027), Macrophages (p < 0.001), T helper cells (p < 0.001), Tcm (p < 0.001), Tgd (p < 0.001), Th1 cell (p < 0.001), Th2 cells (p < 0.001) infiltration levels were significantly higher than those in low SERP1 expression group. But the NK CD56 bright cells (p < 0.001), NK cells (p = 0.016), Mast cells (p = 0.003) infiltration level was significant lower in high SERP1 expression group than low SERP1 expression group.

Moreover, the association of SERP1 expression with markers of mainly relative immune infiltration cells and immune checkpoints of SKCM were explored. The former mainly included.

CD8+ T cell, monocyte, tumor-associated macrophage (TAM), M1 macrophage, M2 macrophage, neutrophils, DC, Th1, Th2, Tfh, Th17 and Treg. The latter mainly included PD-1, PD-L1, CTLA4, LAG3, TIM-3, GZMB, TIGIT and BTLA. We found that SERP1 expression is significantly positive correlated with the most (36 of 41) immune cell markers in SKCM (P < 0.05) (Table 2). Although none of the correlations were high (r < 0.5), but it suggests that SERP1 is extensively involved in regulating immune cell function in SKCM. It is worth noting that, SERP1 expression was positively correlated with numerous immune checkpoint gene markers, including PD-1, PD-L1, PD-L2, CTLA4, LAG3, TIM-3, GZMB, TIGIT (Table 3). These results suggest that SERP1 expression may be associated with the immunotherapeutic effect of SKCM.

**Table 2:** Correlation analysis between SERP1 and relate gene markers of immune cells in SKCM.

**Immune cell**	**Biomarker**	**SERP1**	
		**R**	***P***
CD8+ T cell	CD8A	0.200	<0.001
	CD8B	0.170	<0.001
Monocyte	CD115 (CSF1R)	0.340	<0.001
	CD14	0.240	<0.001
	CD86	0.390	0.001
TAM	CCL2	0.031	<0.001
	CD68	0.043	0.357
	IL10	0.350	<0.001
M1 macrophage	NOS2	0.043	0.351
	IRF5	0.180	<0.001
	PTGS2	0.240	<0.001
M2 macrophage	CD163	0.400	<0.001
	VSIG4	0.320	<0.001
	MS4A4A	0.380	<0.001
Neutrophils	CEACAM8	0.190	<0.001
	CD11b (ITGAM)	0.330	<0.001
	CCR7	0.170	<0.001
Dendritic cell	HLA-DPB1	0.150	<0.001
	HLA-DQB1	0.150	<0.001
	HLA-DRA	0.220	<0.001
	HLA-DPA1	0.150	<0.001
	BDCA-1 (CD1C)	0.230	<0.001
	BDCA-4 (NRP-1)	0.460	<0.001
	CD11C (ITGAX)	0.170	<0.001
Th1	T-bet (TBX21)	0.220	<0.001
	STAT4	0.370	<0.001
	STAT1	0.400	<0.001
	IFN-γ (IFNG)	0.250	<0.001
	TNF-α (TNF)	0.170	<0.001
Th2	GATA3	0.180	<0.001
	STAT6	0.080	0.082
	STAT5A	0.001	0.987
	IL13	0.130	0.005
Tfh	BCL6	0.390	<0.001
	IL21	0.350	<0.001
Th17	STAT3	0.300	<0.001
	IL17A	-0.110	0.019
Treg	FOXP3	0.130	0.004
	CCR8	0.340	<0.001
	STAT5B	0.250	<0.001
	TGFβ (TGFB1)	0.220	<0.001

Abbreviations: SERP1, Stress associated endoplasmic reticulum protein 1; SKCM, Skin Cutaneous Melanoma; TAM, tumor-associated macrophage, TAM.

**Table 3:** Correlation analysis between SERP1 and immune checkpoints in SKCM.

**Immune checkpoints**	**SERP1**	
	**R**	***P***
PD-1 (PDCD1)	0.130	<0.001
PD-L1(CD274)	0.320	<0.001
PD-L2(PDCD1LG2)	0.390	<0.001
CTLA4	0.230	<0.001
LAG3	0.170	<0.001
TIM-3 (HAVCR2)	0.310	<0.001
GZMB	0.180	<0.001
TIGIT	0.260	<0.001
BTLA	0.310	<0.001

Abbreviations: SERP1, Stress associated endoplasmic reticulum protein 1; SKCM, Skin Cutaneous Melanoma.

## DISCUSSION

The ER is a prominent organelle in eukaryotic cells that is involved in regulating calcium homeostasis, the synthesis and folding of secretory and transmembrane proteins, and lipid biosynthesis [14]. Disruption of protein folding in the ER can result from different types of stress, such as inflammatory stimuli, a disruption of calcium homeostasis, nutrient deprivation, an imbalance in redox homeostasis, or an acute increase in protein synthesis [15]. Adverse environmental conditions caused by tumors, such as high metabolic demand, hypoxia, nutrient deprivation, acidosis, and accumulation of reactive oxygen species, can lead to ER stress [16]. This triggers an adaptive response called the unfolded protein response (UPR), aimed at increasing the folding and clearance capacity, thus restoring ER homeostasis [17]. Our understanding of the role of ER stress in various tumors has grown recently [18]. The ER stress response has emerged as a crucial player in initiation, metastasis, prognosis, and immunity in various tumors [19, 20] and is becoming a prevalent target in cancer therapy [21]. SKCM is a highly aggressive and fatal cancer with poor treatment outcomes after progression [22]. Although an association between SKCM and ER has been demonstrated [23], there are few studies of specific genetic markers.

SERP1 is a polypeptide produced during ER stress that protects unfolded proteins from degradation and is associated with poor prognosis in various tumors [12, 13, 24]. Since there had been no clinical studies or basic experiments investigating the effect of SERP1 on SKCM, we conducted a comprehensive bioinformatics exploration via large-scale bioinformatics databases. Our study revealed significantly lower SERP1 levels in patients with SKCM, identified DEGs with the greatest difference in expression between high- and low-SERP1 expression groups and identified transcripts that are highly correlated with SERP1 expression in SKCM. The PPI of SERP1 and GGI of its neighboring genes revealed that these genes are correlated with the response to ER stress and UPR regulation, suggesting that SERP1 participates in ER stress during SKCM tumorigenesis.

In this study, low SERP1 expression was associated with lower OS, PFI, and DSS rates. We also observed significant associations between low SERP1 expression and OS with several patient and clinical parameters including race, age, TNM stage, pathologic stage, absence of radiation therapy, tumor tissue site, melanoma ulceration, and Melanoma Clark Level. Low SERP1 expression correlated with several clinical variables, such as T stage, pathologic stage, Breslow depth, Melanoma ulceration, and radiation therapy. These results illustrate that SERP1 serves an important role in SKCM proliferation, metastasis, and treatment strategy.

The mutation rate of SERP1 in patients with SKCM in this study was 1.1%, with genetic alterations correlated with shorter OS; moreover, results indicated that SERP1 expression level is an independent prognostic factor of OS and DSS for patients with SKCM, suggesting that SERP1 is involved in SKCM progression. However, the results of this study differ from previous findings that high SERP1 expression is associated with poor tumor prognosis [12, 13, 24]. This discrepancy may be related to the dual effects of ER stress. Different levels of ER stress, cell types, and specific tumor microenvironments will lead to different outcomes of the ER stress response [25]. On one hand, excessive or unresolved ER stress can cause cell death. On the other hand, moderate non-lethal ER stress will lead to ER homeostasis recovery and adapt tumor cells to autophagy and apoptosis [26]. Low SERP1 expression in patients with SKCM may be a manifestation of a relatively moderate magnitude of ER stress, thus maintaining the survival of cutaneous melanoma cells.

Early stage SKCM has a better prognosis after surgical resection; however, SKCM tends to subcutaneously metastasize to regional lymph nodes and distant organs, which complicated treatment for advanced stages [27]. Netanely et al. [28] divided melanoma tumors into four subtypes according to different gene expression signatures and validated the classification. They found that the keratin group has the lowest survival rate, significantly higher Breslow depths, and higher pathologic T values. This group is characterized by overexpression of cornification, epidermis development, and keratin-related genes that play a crucial role in forming the outermost skin barrier [29]. This is consistent with the GO and KEGG enrichment analysis of SERP1 in patients with SKCM in our study. Additionally, the predicted genes used in the study to validate the keratin group, including IVL and SPRR1B [28], are consistent with the hub genes of DEGs from cytoHubba and MCODE in our analysis. Thus, keratinization, epidermis development, and cornification related to SERP1 are likely to play an important role in the tumorigenesis and prognosis of SKCM.

SKCM cancer cells and the tumor microenvironment (TME) constitute the SKCM tissue [30]. The TME of SKCM includes the surrounding immune cells, fibroblasts, inflammatory cells, various signaling molecules, and extracellular matrix. The immune cells within the TME and the way they are regulated play an important role in tumorigenesis and progression [31].

Tumor-infiltrating immune cells of the adaptive and innate immune systems infiltrate into the TME and serve a critical role in the modulation of tumor progression [32]. Our results revealed a positive association between SERP1 expression and the infiltration levels of most T cell populations, B cells, macrophages, aDCs, and eosinophils. Conversely, the infiltration levels of NK cells, mast cells, NK CD56 bright cells, pDC and Th17 were negatively correlated with SERP1 expression. Previous studies [33, 34] found that high infiltrates of B cell, T cells were related to a better survival in the SKCM, to some extent, is consistent with our findings.

Additionally, we observed significant differences between infiltrating immune cells in SKCM between the high- and low-SERP1 expression groups. Compared with the low-SERP1 expression group, most T cell populations, B cells, macrophages, aDCs, and eosinophils were present in significantly higher proportions in the high-SERP1 expression group while NK CD56 bright cells, NK cells and Mast cells were significantly lower. The above results agree with previous single-cell-based studies [35–38] of immune infiltrates in melanoma. T cells serve an important role in effective anti-tumor immunity owing to their potent tumor-killing ability [32]. The main function of T helper cells is to amplify the immune response of other immune cells, such as the killing effect of cytotoxic T cells. Th1 cells heighten antigen presentation and are key players in cellular immunity while Th2 cells promote B-cell maturation and enhance the humoral immune response [39]. B cells can perform anti-tumor immune functions by participating in humoral and cellular immunity and acting as antigen-presenting cells [40]. Macrophages and DCs contribute to intrinsic immunity and can regulate tumor progression [41, 42]. Diminished cytotoxic effects and altered expression of pro-inflammatory factors in the tumor microenvironment can impede NK cell function [43] and can lead to immune escape [44]. Mast cells are derived from bone marrow hematopoietic progenitor cells and are broadly distributed throughout the body. Early mast cell infiltration is widespread in solid tumors, especially in malignant melanoma [45]. Mast cells can release various proangiogenic factors, such as VEGF, FGF-2, PDGF, IL-6, tryptase, and chymase, to promote tumor angiogenesis and induce neovascularization. It can also release matrix metalloproteinases to promote tumor invasiveness [46]. Our results of immune infiltration combined with the above reveal that SERP1 is involved in both the innate and adaptive immune responses to SKCM. This, to some extent, implies that differences in immune infiltration produced by SERP1 expression profoundly affect the prognosis of patients with SKCM.

Moreover, our study found that SERP1 expression levels positively correlated with most immune cell markers suggesting the positive role of SERP1 in modulating tumor immunology in SKCM. Interestingly, the correlation between the gene markers of M1 macrophage and SERP1 expression was lower than that of M2 macrophage, implying SERP1 may be involved in the polarization of TAM. Further, our result confirmed that SERP1 expression in SKCM patients was positively correlated with numerous immune checkpoint gene markers. Given that multiple targeted immune checkpoint blockade therapies, especially PD-1 [47, 48] and CTLA4 [49, 50], have shown significant efficacy in melanoma treatment, SERP1 expression levels in SKCM patients may also influence the efficacy of immunotherapy.

In conclusion, the expression of SERP1, an ER response-related gene, is reduced in patients with SKCM. SERP1 expression level is associated with diverse clinical variables. Decreased SERP1 expression results in decreased survival of patients with SKCM with some clinical features and is an independent risk factor for poor prognosis in SKCM. Thus, it can serve as a biomarker with the capacity to predict prognosis in patients with SKCM. Genes closely associated with SERP1 are primarily involved in keratinization, epidermis development, and cornification, which play a pivotal role in the poor prognosis and tumorigenesis of SKCM. SERP1 expression is also significantly associated with the infiltration of multiple immune cells and immune checkpoints, involved in both the innate and adaptive immune systems of SKCM, and participates in the construction of the SKCM TME. We hope that further study on SERP1 in SKCM will confirm its potential for clinical application. It is expected that further exploration of how SERP1 is involved in ER stress and affects the TME will shed light on immunotherapy options for SKCM.

## MATERIALS AND METHODS

### Gene expression and clinical data

We obtained the mRNA expression and clinical data from TCGA (https://cancergenome.nih.gov). The data for the corresponding 813 normal tissue samples were obtained from the GTEx (https://gtexportal.org/). Samples with “0” values for gene expression or with inadequate prognostic information were excluded.

RNA-sequencing data in Fragments Per Kilobase per Million format (FPKM) were converted and normalized by the Toil [51] process as transcripts per million (PTM) reads and log2 transformed for further analysis. The clinical characteristics of the 472 patients included in the study are summarized in Supplementary Table 1. Location and qualitative data of SERP1 protein in SKCM and normal tissues were evaluated by the Human Protein Atlas (https://www.proteinatlas.org/) [52].

### DEGs analysis between SKCM patients with high and low SERP1 expression

We performed a “DESeq2” analysis [53] in R to identify DEGs between SERP1-high and SERP1-low SKCM patients identified by unpaired Student’s t-test. Thresholds were set as an adjusted P < 0.05 and absolute log-fold change > 3. Identified genes were analyzed and presented as volcano plots. The top 15 up- or down-regulated genes were presented as heat maps. All data visualization was achieved using the “ggplot2” package in R. We set a relatively high threshold (log-fold change > 3) with the aim of selecting the DEGs with the greatest degree of change associated with SERP1-high and SERP1-low expression. The top 15 DEGs were selected to further screen the genes most associated with SERP1-high and SERP1-low expression.

### SERP1 correlation analysis in patients with SKCM

We performed correlation analysis between SERP1 and other RNAs in patients with SKCM using TCGA data via the “stat” package in R. Pearson correlation coefficients were calculated and the top 50 genes most positively and negatively associated with SERP1 were filtered to construct heat maps using the “ggplot2” package in R.

### PPI network and GGI network analysis

To collect and integrate potential protein interactions with SERP1, we searched the STRING database (https://string-db.org/) [54] and conducted a PPI network analysis. A confidence score > 0.7 was set as the significance threshold. GeneMANIA (http://www.genemania.org) [55] uses extensive genomics and proteomics data to discover functionally similar genes. The database is used to generate hypotheses about gene function, analyze gene lists, and prioritize genes for functional analysis. We searched for SERP1 in this database to predict the gene–gene interaction (GGI) network.

### Functional enrichment analysis of DEGs between patients with SKCM with high- and low-SERP1 expression

The “clusterProfiler” [56] and “org.Hs.eg.db” packages of R and the “ClueGO” app of Cytoscape (v3.8.2) were used to conduct GO function and KEGG pathway enrichment analyses for statistically significant DEGs. A p-value < 0.01 was set as the cut-off threshold for GO and KEGG pathway enrichment analyses. The results were presented as a bar plot via the “ggplot2” package in R. Additionally, we also imported the DEGs into the STRING database to build a PPI network map and completed the GO and KEGG signaling pathway enrichment analyses using the ClueGO plugin of Cytoscape. The MCODE and cytoHubba plugins were used to identify key modules. The top 10 nodes ranked by MCC of cytoHubba and modules with MCODE score = 30 were presented.

### Gene set enrichment analysis

We performed GSEA via the “clusterProfiler” [56] package to determine the biological pathway differences between high- and low-SERP1 groups. Pathways with a false discovery rate (FDR) < 0.25 and an adjusted p-value < 0.05 were considered to be remarkably changed. Gene set permutation was performed 1,000 times for each analysis.

### cBioPortal

We searched and downloaded mutation information and corresponding clinical data from cBioPortal (https://www.cbioportal.org/) [57], a comprehensive database that provides visual and multidimensional cancer genomics data.

### Kaplan–Meier analysis

We conducted Kaplan–Meier analysis via the “survival” package to compare the OS, DSS, and PFI rates between the high- and low-SERP1 gene expression groups. Subgroup Kaplan–Meier analysis of OS was also performed based on different clinical variables. The p-value was determined by Cox regression analysis and the survival curves were visualized via the “survminer” package.

### Correlation between SERP1 expression and immune infiltration in SKCM

We used the gene set variation analysis package [58] to investigate the correlation between the SERP1 expression and tumor-infiltrating immune cells in patients with SKCM. Twenty-four types of immune cells were included in the analysis: T cells, aDC (activated DC), B cells, CD8 T cells, cytotoxic cells, DC, eosinophils, iDC (immature DC), macrophages, mast cells, neutrophils, NK CD56 bright cells, NK CD56 dim cells, NK cells, pDC (plasmacytoid DC), T helper cells, Tcm (central memory T cells), Tem (effector memory T cells), Tfh (T follicular helper cells), Tgd (gamma delta T cells), Th1 cells, Th17 cells, Th2 cells, and Treg (regulatory T cells). The association of SERP1 with immune cell markers as well as immune checkpoints were also explored. Spearman’s correlation was used to evaluate the correlation of gene expression with p-values < 0.05 considered statistically significant.

### Statistical analysis

R software was used to perform statistical analyses in this study (version 3.6.3). The “ggplot2” package was used to present SERP1 gene expression as dot plots in patients with pan-cancer and SKCM. The median method of gene expression was selected for cutoff values. SERP1 expression in patients with SKCM with different clinical characteristics was analyzed using the Chi-squared test, Fisher test, and Wilcoxon rank sum test depending on the situation. The “survival” package was used to analyze the effect of SERP1 on survival with other clinical characteristics in SKCM patients via univariate and multivariate Cox regression. The predictive nomogram of 1-, 3-, and 5-year OS, DSS and PFI with SERP1 expression for SKCM patients and other relevant clinical parameters was constructed via a stepwise Cox regression model through the “rms” and “survival” packages. The ROC curve, C-index and calibration curve were used to verify the reliability of nomograms. ROC curves were analyzed and the areas under the ROC curve (AUC) were calculated via the timeROC (version 0.4) package of R software. The calibration curves were analyzed and plotted via rms (version 6.2-0) and survival (version 3.2-10) package of R software. The number of samples per group for our calibration curves is set to 40, the number of repetitions is 200, and the boot method is used.

## Supplementary Material

Supplementary Figures

Supplementary Tables

## References

[1] Bray F, Ferlay J, Soerjomataram I, Siegel RL, Torre LA, Jemal A. Global cancer statistics 2018: GLOBOCAN estimates of incidence and mortality worldwide for 36 cancers in 185 countries. CA Cancer J Clin. 2018; 68:394–424. 10.3322/caac.21492 30207593

[2] Viale PH. The American Cancer Society’s Facts and Figures: 2020 Edition. J Adv Pract Oncol. 2020; 11:135–36. 10.6004/jadpro.2020.11.2.1 33532112 PMC7848816

[3] Bertrand JU, Steingrimsson E, Jouenne F, Bressac-de Paillerets B, Larue L. Melanoma Risk and Melanocyte Biology. Acta Derm Venereol. 2020; 100:adv00139. 10.2340/00015555-3494 32346747 PMC9189750

[4] Yang K, Oak AS, Slominski RM, Brożyna AA, Slominski AT. Current Molecular Markers of Melanoma and Treatment Targets. Int J Mol Sci. 2020; 21:3535. 10.3390/ijms21103535 32429485 PMC7278971

[5] Guy GP Jr, Ekwueme DU, Tangka FK, Richardson LC. Melanoma treatment costs: a systematic review of the literature, 1990-2011. Am J Prev Med. 2012; 43:537–45. 10.1016/j.amepre.2012.07.031 23079178 PMC4495902

[6] Gershenwald JE, Scolyer RA, Hess KR, Sondak VK, Long GV, Ross MI, Lazar AJ, Faries MB, Kirkwood JM, McArthur GA, Haydu LE, Eggermont AM, Flaherty KT, et al, and for members of the American Joint Committee on Cancer Melanoma Expert Panel and the International Melanoma Database and Discovery Platform. Melanoma staging: Evidence-based changes in the American Joint Committee on Cancer eighth edition cancer staging manual. CA Cancer J Clin. 2017; 67:472–92. 10.3322/caac.21409 29028110 PMC5978683

[7] Cachia AR, Indsto JO, McLaren KM, Mann GJ, Arends MJ. CDKN2A mutation and deletion status in thin and thick primary melanoma. Clin Cancer Res. 2000; 6:3511–15. 10999737

[8] Kanemaru H, Fukushima S, Yamashita J, Honda N, Oyama R, Kakimoto A, Masuguchi S, Ishihara T, Inoue Y, Jinnin M, Ihn H. The circulating microRNA-221 level in patients with malignant melanoma as a new tumor marker. J Dermatol Sci. 2011; 61:187–93. 10.1016/j.jdermsci.2010.12.010 21273047

[9] Gerami P, Cook RW, Wilkinson J, Russell MC, Dhillon N, Amaria RN, Gonzalez R, Lyle S, Johnson CE, Oelschlager KM, Jackson GL, Greisinger AJ, Maetzold D, et al. Development of a prognostic genetic signature to predict the metastatic risk associated with cutaneous melanoma. Clin Cancer Res. 2015; 21:175–83. 10.1158/1078-0432.CCR-13-3316 25564571

[10] Armand-Labit V, Meyer N, Casanova A, Bonnabau H, Platzer V, Tournier E, Sansas B, Verdun S, Thouvenot B, Hilselberger B, Doncescu A, Lamant L, Lacroix-Triki M, et al. Identification of a Circulating MicroRNA Profile as a Biomarker of Metastatic Cutaneous Melanoma. Acta Derm Venereol. 2016; 96:29–34. 10.2340/00015555-2156 26039581

[11] Yamaguchi A, Hori O, Stern DM, Hartmann E, Ogawa S, Tohyama M. Stress-associated endoplasmic reticulum protein 1 (SERP1)/Ribosome-associated membrane protein 4 (RAMP4) stabilizes membrane proteins during stress and facilitates subsequent glycosylation. J Cell Biol. 1999; 147:1195–204. 10.1083/jcb.147.6.1195 10601334 PMC2168098

[12] Ma Q, Wu X, Wu J, Liang Z, Liu T. SERP1 is a novel marker of poor prognosis in pancreatic ductal adenocarcinoma patients via anti-apoptosis and regulating SRPRB/NF-κB axis. Int J Oncol. 2017; 51:1104–14. 10.3892/ijo.2017.4111 28902358 PMC5592859

[13] Mucaj V, Lee SS, Skuli N, Giannoukos DN, Qiu B, Eisinger-Mathason TS, Nakazawa MS, Shay JE, Gopal PP, Venneti S, Lal P, Minn AJ, Simon MC, Mathew LK. MicroRNA-124 expression counteracts pro-survival stress responses in glioblastoma. Oncogene. 2015; 34:2204–14. 10.1038/onc.2014.168 24954504 PMC4275412

[14] Jain BP. An Overview of Unfolded Protein Response Signaling and Its Role in Cancer. Cancer Biother Radiopharm. 2017; 32:275–81. 10.1089/cbr.2017.2309 29053418

[15] Urra H, Dufey E, Avril T, Chevet E, Hetz C. Endoplasmic Reticulum Stress and the Hallmarks of Cancer. Trends Cancer. 2016; 2:252–62. 10.1016/j.trecan.2016.03.007 28741511

[16] Song M, Cubillos-Ruiz JR. Endoplasmic Reticulum Stress Responses in Intratumoral Immune Cells: Implications for Cancer Immunotherapy. Trends Immunol. 2019; 40:128–41. 10.1016/j.it.2018.12.001 30612925

[17] Vanacker H, Vetters J, Moudombi L, Caux C, Janssens S, Michallet MC. Emerging Role of the Unfolded Protein Response in Tumor Immunosurveillance. Trends Cancer. 2017; 3:491–505. 10.1016/j.trecan.2017.05.005 28718404

[18] Dufey E, Sepúlveda D, Rojas-Rivera D, Hetz C. Cellular mechanisms of endoplasmic reticulum stress signaling in health and disease. 1. An overview. Am J Physiol Cell Physiol. 2014; 307:C582–94. 10.1152/ajpcell.00258.2014 25143348

[19] Lebeaupin C, Yong J, Kaufman RJ. The Impact of the ER Unfolded Protein Response on Cancer Initiation and Progression: Therapeutic Implications. Adv Exp Med Biol. 2020; 1243:113–31. 10.1007/978-3-030-40204-4_8 32297215 PMC7243802

[20] Mohamed E, Cao Y, Rodriguez PC. Endoplasmic reticulum stress regulates tumor growth and anti-tumor immunity: a promising opportunity for cancer immunotherapy. Cancer Immunol Immunother. 2017; 66:1069–78. 10.1007/s00262-017-2019-6 28577085 PMC5700458

[21] Wang M, Law ME, Castellano RK, Law BK. The unfolded protein response as a target for anticancer therapeutics. Crit Rev Oncol Hematol. 2018; 127:66–79. 10.1016/j.critrevonc.2018.05.003 29891114

[22] MacKie RM, Hauschild A, Eggermont AM. Epidemiology of invasive cutaneous melanoma. Ann Oncol. 2009 (Suppl 6); 20:vi1–7. 10.1093/annonc/mdp252 19617292 PMC2712590

[23] Manga P, Choudhury N. The unfolded protein and integrated stress response in melanoma and vitiligo. Pigment Cell Melanoma Res. 2021; 34:204–11. 10.1111/pcmr.12947 33215847

[24] Liu W, Wang D, Wang X, Liu P, Yan M. hsa_circ_0085539 Promotes Osteosarcoma Progression by Regulating miR-526b-5p and SERP1. Mol Ther Oncolytics. 2020; 19:163–77. 10.1016/j.omto.2020.09.009 33209976 PMC7649436

[25] Chen X, Cubillos-Ruiz JR. Endoplasmic reticulum stress signals in the tumour and its microenvironment. Nat Rev Cancer. 2021; 21:71–88. 10.1038/s41568-020-00312-2 33214692 PMC7927882

[26] Walter P, Ron D. The unfolded protein response: from stress pathway to homeostatic regulation. Science. 2011; 334:1081–86. 10.1126/science.1209038 22116877

[27] Houghton AN, Polsky D. Focus on melanoma. Cancer Cell. 2002; 2:275–78. 10.1016/s1535-6108(02)00161-7 12398891

[28] Netanely D, Leibou S, Parikh R, Stern N, Vaknine H, Brenner R, Amar S, Factor RH, Perluk T, Frand J, Nizri E, Hershkovitz D, Zemser-Werner V, et al. Classification of node-positive melanomas into prognostic subgroups using keratin, immune, and melanogenesis expression patterns. Oncogene. 2021; 40:1792–805. 10.1038/s41388-021-01665-0 33564068 PMC7946641

[29] Eckhart L, Tschachler E. Control of cell death-associated danger signals during cornification prevents autoinflammation of the skin. Exp Dermatol. 2018; 27:884–91. 10.1111/exd.13700 29862564

[30] Romano V, Belviso I, Venuta A, Ruocco MR, Masone S, Aliotta F, Fiume G, Montagnani S, Avagliano A, Arcucci A. Influence of Tumor Microenvironment and Fibroblast Population Plasticity on Melanoma Growth, Therapy Resistance and Immunoescape. Int J Mol Sci. 2021; 22:5283. 10.3390/ijms22105283 34067929 PMC8157224

[31] Spano D, Zollo M. Tumor microenvironment: a main actor in the metastasis process. Clin Exp Metastasis. 2012; 29:381–95. 10.1007/s10585-012-9457-5 22322279

[32] Zhang Y, Zhang Z. The history and advances in cancer immunotherapy: understanding the characteristics of tumor-infiltrating immune cells and their therapeutic implications. Cell Mol Immunol. 2020; 17:807–21. 10.1038/s41423-020-0488-6 32612154 PMC7395159

[33] Azimi F, Scolyer RA, Rumcheva P, Moncrieff M, Murali R, McCarthy SW, Saw RP, Thompson JF. Tumor-infiltrating lymphocyte grade is an independent predictor of sentinel lymph node status and survival in patients with cutaneous melanoma. J Clin Oncol. 2012; 30:2678–83. 10.1200/JCO.2011.37.8539 22711850

[34] Selitsky SR, Mose LE, Smith CC, Chai S, Hoadley KA, Dittmer DP, Moschos SJ, Parker JS, Vincent BG. Prognostic value of B cells in cutaneous melanoma. Genome Med. 2019; 11:36. 10.1186/s13073-019-0647-5 31138334 PMC6540526

[35] Tirosh I, Izar B, Prakadan SM, Wadsworth MH 2nd, Treacy D, Trombetta JJ, Rotem A, Rodman C, Lian C, Murphy G, Fallahi-Sichani M, Dutton-Regester K, Lin JR, et al. Dissecting the multicellular ecosystem of metastatic melanoma by single-cell RNA-seq. Science. 2016; 352:189–96. 10.1126/science.aad0501 27124452 PMC4944528

[36] Li H, van der Leun AM, Yofe I, Lubling Y, Gelbard-Solodkin D, van Akkooi AC, van den Braber M, Rozeman EA, Haanen JB, Blank CU, Horlings HM, David E, Baran Y, et al. Dysfunctional CD8 T Cells Form a Proliferative, Dynamically Regulated Compartment within Human Melanoma. Cell. 2020; 181:747. 10.1016/j.cell.2020.04.017 32359441

[37] Jerby-Arnon L, Shah P, Cuoco MS, Rodman C, Su MJ, Melms JC, Leeson R, Kanodia A, Mei S, Lin JR, Wang S, Rabasha B, Liu D, et al. A Cancer Cell Program Promotes T Cell Exclusion and Resistance to Checkpoint Blockade. Cell. 2018; 175:984–97.e24. 10.1016/j.cell.2018.09.006 30388455 PMC6410377

[38] Sade-Feldman M, Yizhak K, Bjorgaard SL, Ray JP, de Boer CG, Jenkins RW, Lieb DJ, Chen JH, Frederick DT, Barzily-Rokni M, Freeman SS, Reuben A, Hoover PJ, et al. Defining T Cell States Associated with Response to Checkpoint Immunotherapy in Melanoma. Cell. 2019; 176:404. 10.1016/j.cell.2018.12.034 30633907 PMC6647017

[39] Dong C. Cytokine Regulation and Function in T Cells. Annu Rev Immunol. 2021; 39:51–76. 10.1146/annurev-immunol-061020-053702 33428453

[40] Tsou P, Katayama H, Ostrin EJ, Hanash SM. The Emerging Role of B Cells in Tumor Immunity. Cancer Res. 2016; 76:5597–601. 10.1158/0008-5472.CAN-16-0431 27634765

[41] Geissmann F, Manz MG, Jung S, Sieweke MH, Merad M, Ley K. Development of monocytes, macrophages, and dendritic cells. Science. 2010; 327:656–61. 10.1126/science.1178331 20133564 PMC2887389

[42] Long KB, Collier AI, Beatty GL. Macrophages: Key orchestrators of a tumor microenvironment defined by therapeutic resistance. Mol Immunol. 2019; 110:3–12. 10.1016/j.molimm.2017.12.003 29273393 PMC6008174

[43] Paul S, Lal G. The Molecular Mechanism of Natural Killer Cells Function and Its Importance in Cancer Immunotherapy. Front Immunol. 2017; 8:1124. 10.3389/fimmu.2017.01124 28955340 PMC5601256

[44] Böttcher JP, Bonavita E, Chakravarty P, Blees H, Cabeza-Cabrerizo M, Sammicheli S, Rogers NC, Sahai E, Zelenay S, Reis e Sousa C. NK Cells Stimulate Recruitment of cDC1 into the Tumor Microenvironment Promoting Cancer Immune Control. Cell. 2018; 172:1022–37.e14. 10.1016/j.cell.2018.01.004 29429633 PMC5847168

[45] Molderings GJ, Zienkiewicz T, Homann J, Menzen M, Afrin LB. Risk of solid cancer in patients with mast cell activation syndrome: Results from Germany and USA. F1000Res. 2017; 6:1889. 10.12688/f1000research.12730.1 29225779 PMC5710302

[46] Komi DE, Redegeld FA. Role of Mast Cells in Shaping the Tumor Microenvironment. Clin Rev Allergy Immunol. 2020; 58:313–25. 10.1007/s12016-019-08753-w 31256327 PMC7244463

[47] Topalian SL, Hodi FS, Brahmer JR, Gettinger SN, Smith DC, McDermott DF, Powderly JD, Carvajal RD, Sosman JA, Atkins MB, Leming PD, Spigel DR, Antonia SJ, et al. Safety, activity, and immune correlates of anti-PD-1 antibody in cancer. N Engl J Med. 2012; 366:2443–54. 10.1056/NEJMoa1200690 22658127 PMC3544539

[48] Eggermont AM, Robert C, Ribas A. The new era of adjuvant therapies for melanoma. Nat Rev Clin Oncol. 2018; 15:535–36. 10.1038/s41571-018-0048-5 29849093

[49] Hodi FS, O’Day SJ, McDermott DF, Weber RW, Sosman JA, Haanen JB, Gonzalez R, Robert C, Schadendorf D, Hassel JC, Akerley W, van den Eertwegh AJ, Lutzky J, et al. Improved survival with ipilimumab in patients with metastatic melanoma. N Engl J Med. 2010; 363:711–23. 10.1056/NEJMoa1003466 20525992 PMC3549297

[50] Schadendorf D, Hodi FS, Robert C, Weber JS, Margolin K, Hamid O, Patt D, Chen TT, Berman DM, Wolchok JD. Pooled Analysis of Long-Term Survival Data From Phase II and Phase III Trials of Ipilimumab in Unresectable or Metastatic Melanoma. J Clin Oncol. 2015; 33:1889–94. 10.1200/JCO.2014.56.2736 25667295 PMC5089162

[51] Vivian J, Rao AA, Nothaft FA, Ketchum C, Armstrong J, Novak A, Pfeil J, Narkizian J, Deran AD, Musselman-Brown A, Schmidt H, Amstutz P, Craft B, et al. Toil enables reproducible, open source, big biomedical data analyses. Nat Biotechnol. 2017; 35:314–16. 10.1038/nbt.3772 28398314 PMC5546205

[52] Uhlén M, Fagerberg L, Hallström BM, Lindskog C, Oksvold P, Mardinoglu A, Sivertsson Å, Kampf C, Sjöstedt E, Asplund A, Olsson I, Edlund K, Lundberg E, et al. Proteomics. Tissue-based map of the human proteome. Science. 2015; 347:1260419. 10.1126/science.1260419 25613900

[53] Love MI, Huber W, Anders S. Moderated estimation of fold change and dispersion for RNA-seq data with DESeq2. Genome Biol. 2014; 15:550. 10.1186/s13059-014-0550-8 25516281 PMC4302049

[54] Szklarczyk D, Gable AL, Lyon D, Junge A, Wyder S, Huerta-Cepas J, Simonovic M, Doncheva NT, Morris JH, Bork P, Jensen LJ, Mering CV. STRING v11: protein-protein association networks with increased coverage, supporting functional discovery in genome-wide experimental datasets. Nucleic Acids Res. 2019; 47:D607–13. 10.1093/nar/gky1131 30476243 PMC6323986

[55] Warde-Farley D, Donaldson SL, Comes O, Zuberi K, Badrawi R, Chao P, Franz M, Grouios C, Kazi F, Lopes CT, Maitland A, Mostafavi S, Montojo J, et al. The GeneMANIA prediction server: biological network integration for gene prioritization and predicting gene function. Nucleic Acids Res. 2010; 38:W214–20. 10.1093/nar/gkq537 20576703 PMC2896186

[56] Yu G, Wang LG, Han Y, He QY. clusterProfiler: an R package for comparing biological themes among gene clusters. OMICS. 2012; 16:284–87. 10.1089/omi.2011.0118 22455463 PMC3339379

[57] Gao J, Aksoy BA, Dogrusoz U, Dresdner G, Gross B, Sumer SO, Sun Y, Jacobsen A, Sinha R, Larsson E, Cerami E, Sander C, Schultz N. Integrative analysis of complex cancer genomics and clinical profiles using the cBioPortal. Sci Signal. 2013; 6:pl1. 10.1126/scisignal.2004088 23550210 PMC4160307

[58] Hänzelmann S, Castelo R, Guinney J. GSVA: gene set variation analysis for microarray and RNA-seq data. BMC Bioinformatics. 2013; 14:7. 10.1186/1471-2105-14-7 23323831 PMC3618321

