# Supplementary material for: Low expression of endoplasmic reticulum stress-related gene SERP1 is associated with poor prognosis and immune infiltration in skin cutaneous melanoma
Source: Aging (Albany NY). 2021 Oct 5;13(19):23036–71. doi: 10.18632/aging.203594 (PMC8544316; doi:10.18632/aging.203594)
Supplement: Supplementary Figures [file aging-13-203594-s001.pdf]

SUPPLEMENTARY FIGURES

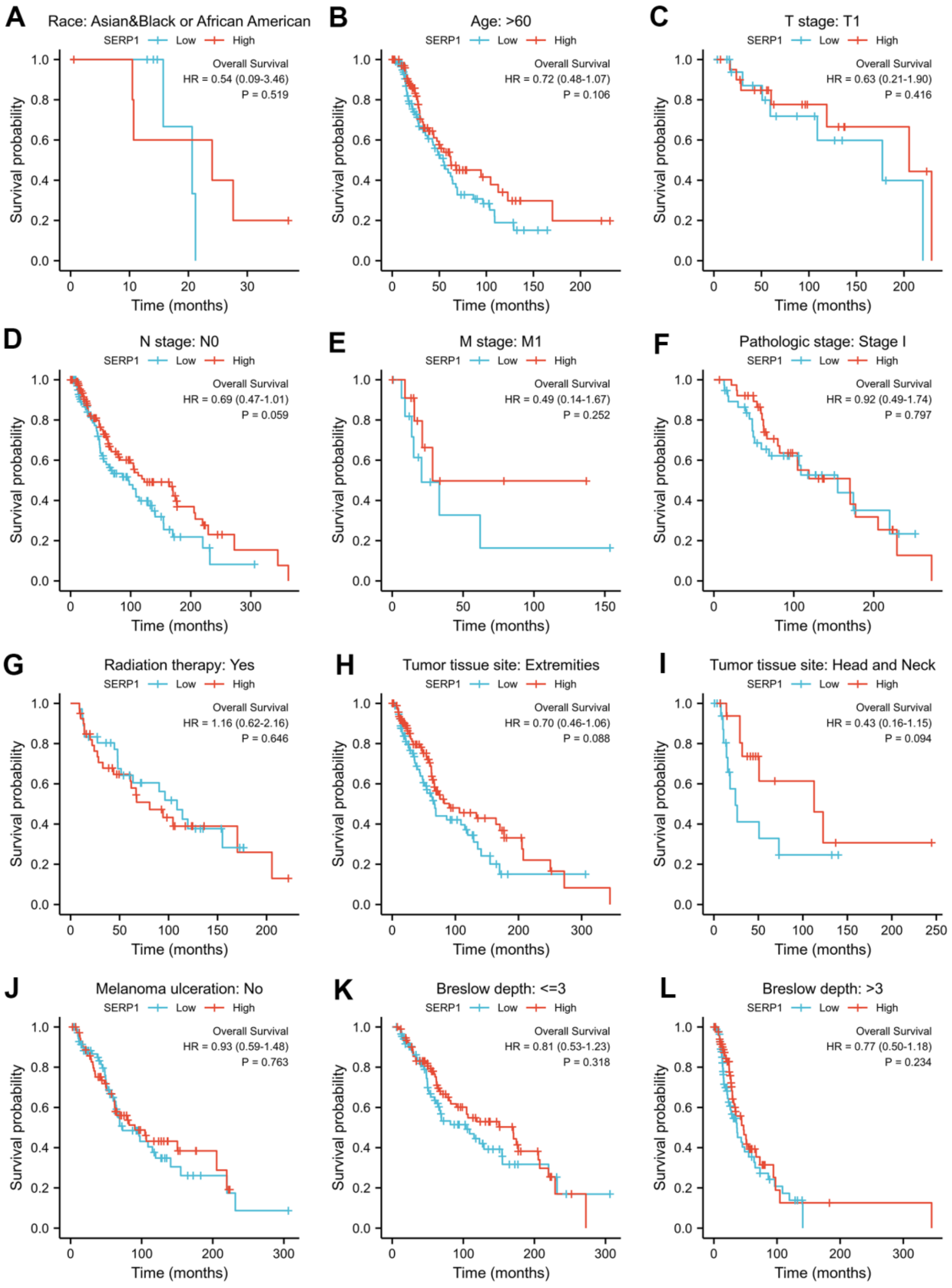

Supplementary Figure 1. The subgroups that high or low SERP1 expression did not show statistical significance on OS. OS Kaplan-Meier curve without statistical significance for (**A**) Asian and Black or African American, (**B**) Age > 60, (**C**) T stage (T1), (**D**) N stage (N0), (**E**) M stage (M1), (**F**) Pathologic Stage (Stage I), (**G**) Radiation therapy Yes, (**H**) Tumor tissue site Extremities, (**I**) Tumor tissue site Head and neck, (**J**) Melanoma ulceration No, (**K**) Breslow depth ≤ 3, (**L**) Breslow depth>3.

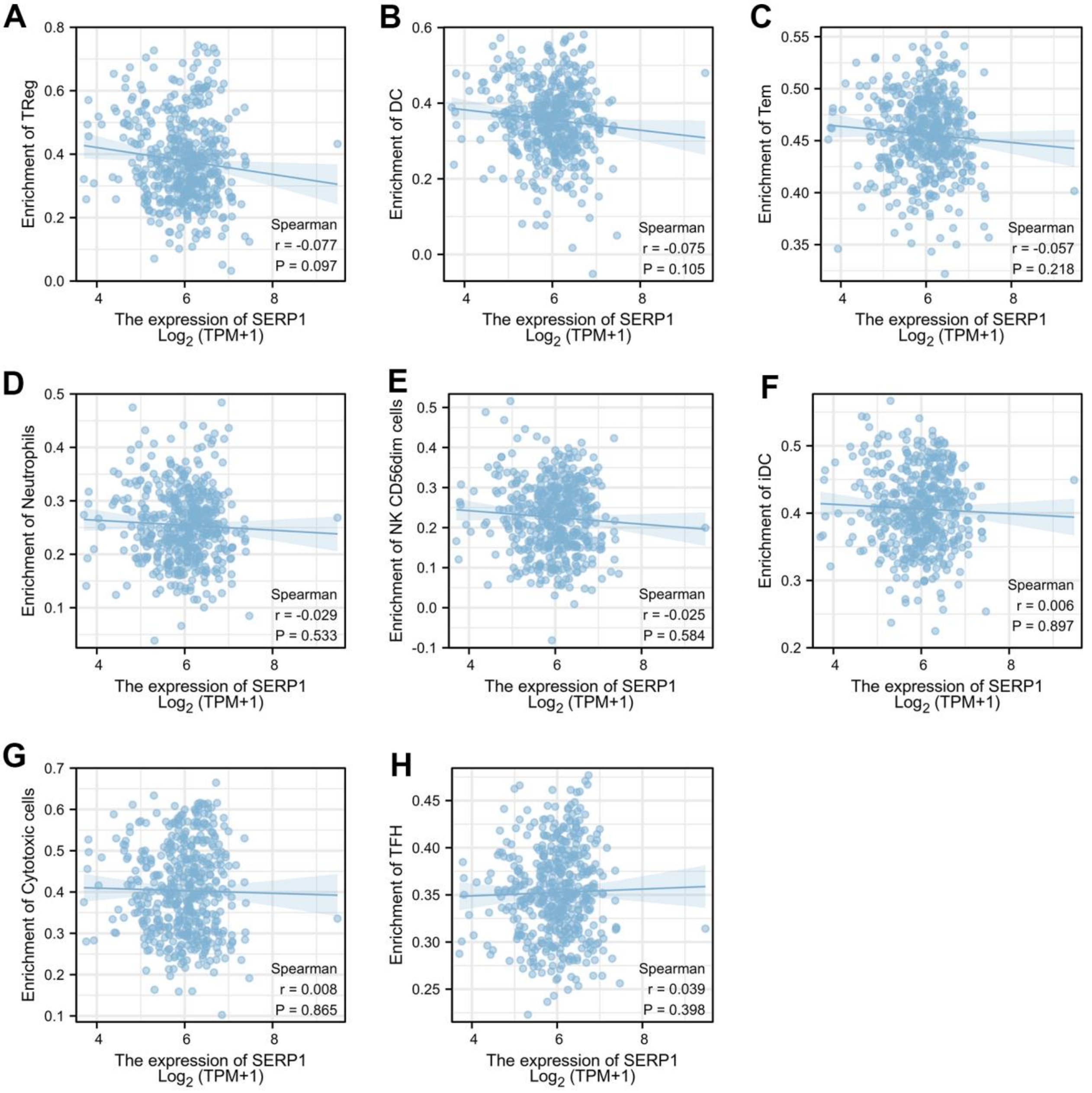

Supplementary Figure 2. Correlation of SERP1 and immune cell infiltration in SKCM. The (**A**) Treg, (**B**) DC, (**C**) Tem, (**D**) Neutrophils, (**E**) NK CD56dim cells, (**F**) iDC, (**G**) Cytotoxic cells, (**H**) TFH showed no significant correlation with SERP1.
