# Supplementary material for: Low expression of endoplasmic reticulum stress-related gene SERP1 is associated with poor prognosis and immune infiltration in skin cutaneous melanoma
Source: Aging (Albany NY). 2021 Oct 5;13(19):23036–71. doi: 10.18632/aging.203594 (PMC8544316; doi:10.18632/aging.203594)
Supplement: Supplementary Tables [file aging-13-203594-s002.pdf]

SUPPLEMENTARY TABLES

Supplementary Table 1. TCGA SKCM patient characteristics.

Characteristic	Levels	Overall
n		471
Gender, n (%)	Female	179 (38%)
	Male	292 (62%)
Age, n (%)	<=60	252 (54.4%)
	>60	211 (45.6%)
Race, n (%)	Asian	12 (2.6%)
	Black or African American	1 (0.2%)
	White	448 (97.2%)
Weight, n (%)	<=70	77 (29.7%)
	>70	182 (70.3%)
Height, n (%)	< 170	118 (46.5%)
	>=170	136 (53.5%)
BMI, n (%)	<=25	84 (33.5%)
	>25	167 (66.5%)
T stage, n (%)	T1	41 (11.3%)
	T2	79 (21.7%)
	T3	91 (25%)
	T4	153 (42%)
N stage, n (%)	N0	235 (56.8%)
	N1	74 (17.9%)
	N2	49 (11.8%)
	N3	56 (13.5%)
M stage, n (%)	M0	418 (94.4%)
	M1	25 (5.6%)
Pathologic stage, n (%)	Stage I	77 (18.7%)
	Stage II	140 (34%)
	Stage III	171 (41.5%)
	Stage IV	24 (5.8%)
Radiation therapy, n (%)	No	383 (82.5%)
	Yes	81 (17.5%)
Tumor tissue site, n (%)	Extremities	197 (47%)
	Trunk	171 (40.8%)
	Head and Neck	38 (9.1%)
	Other Specify	13 (3.1%)
Melanoma ulceration, n (%)	No	147 (46.8%)
	Yes	167 (53.2%)
Melanoma Clark level, n (%)	I	6 (1.9%)
	II	18 (5.6%)
	III	77 (23.9%)
	IV	168 (52.2%)
	V	53 (16.5%)
Breslow depth, n (%)	<=3	185 (51.4%)
	>3	175 (48.6%)
Age, median (IQR)		58 (48, 71)

Abbreviations: SERP1, Stress associated endoplasmic reticulum protein 1; SKCM, Skin Cutaneous Melanoma; IQR, interquartile range. TCGA, The Cancer Genome Atlas; BMI, Body Mass Index.

Supplementary Table 2. Top up and down 15 items of differential expressed genes of SERP1.

Gene_name	Gene_id	Gene_biotype	log2foldchange	lfcse	Stat	pvalue	padj
UP							
PASD1	ENSG00000166049	protein_coding	69.83688	4.640308	0.487069	9.527005	1.62E-21
SMR3B	ENSG00000171201	protein_coding	3.471793	4.547842	0.97397	4.669388	3.02E-06
FOXR2	ENSG00000189299	protein_coding	16.68608	4.371976	0.446959	9.781607	1.35E-22
MAEL	ENSG00000143194	protein_coding	81.48709	4.281782	0.310803	13.77653	3.53E-43
LINC00200	ENSG00000229205	lncRNA	5.463603	4.215688	0.522708	8.065098	7.32E-16
OTOR	ENSG00000125879	protein_coding	19.10208	4.166952	0.649502	6.415615	1.40E-10
CT45A1	ENSG00000268940	protein_coding	10.08509	4.113013	0.609996	6.742684	1.55E-11
AL354685.1	ENSG00000233887	processed_pseudogene	8.446124	4.013919	0.705495	5.68951	1.27E-08
SLITRK1	ENSG00000178235	protein_coding	24.69851	3.989265	0.336024	11.87197	1.66E-32
LINC01203	ENSG00000226985	lncRNA	4.358567	3.974891	0.435579	9.125535	7.14E-20
SAGE1	ENSG00000181433	protein_coding	21.23367	3.946295	0.456596	8.642863	5.48E-18
AC004674.1	ENSG00000235592	unprocessed_pseudogene	1.883181	3.786196	0.706633	5.358079	8.41E-08
FAR2P1	ENSG00000180178	transcribed_unprocessed_pseudogene	45.99805	3.684811	0.393731	9.358707	8.07E-21
BX119904.2	ENSG00000230159	lncRNA	7.019114	3.550922	0.666204	5.33008	9.82E-08
HTN3	ENSG00000205649	protein_coding	134.4259	3.549214	0.505356	7.023189	2.17E-12
DOWN							
KRTDAP	ENSG00000188508	protein_coding	924.0061	-4.52021	0.380391	-11.8831	1.45E-32
CDSN	ENSG00000204539	protein_coding	39.63	-4.52433	0.448062	-10.0976	5.66E-24
KRT71	ENSG00000139648	protein_coding	3.876285	-4.6771	0.585631	-7.98642	1.39E-15
SPRR2E	ENSG00000203785	protein_coding	578.3245	-4.68414	0.456065	-10.2708	9.54E-25
LINC01527	ENSG00000224308	lncRNA	16.24596	-4.71413	0.549555	-8.57809	9.65E-18
SPRR2G	ENSG00000159516	protein_coding	324.7711	-4.73287	0.501736	-9.43299	3.99E-21
KRT1	ENSG00000167768	protein_coding	6338.582	-4.75016	0.415758	-11.4253	3.13E-30
KRT16	ENSG00000186832	protein_coding	12867.72	-4.76411	0.39794	-11.9719	4.98E-33
LCE2B	ENSG00000159455	protein_coding	38.90983	-4.82879	0.889447	-5.42898	5.67E-08
CALML5	ENSG00000178372	protein_coding	926.9186	-4.8295	0.495511	-9.74649	1.91E-22
KRT6C	ENSG00000170465	protein_coding	5147.085	-4.89758	0.474097	-10.3303	5.14E-25
WFDC12	ENSG00000168703	protein_coding	54.46971	-4.99446	0.514118	-9.71461	2.61E-22
LCE3E	ENSG00000185966	protein_coding	63.57593	-5.16652	0.631301	-8.18393	2.75E-16
LORICRIN	ENSG00000203782	protein_coding	229.1628	-5.23657	0.489006	-10.7086	9.28E-27
CASP14	ENSG00000105141	protein_coding	734.9536	-5.77555	0.459991	-12.5558	3.69E-36

Abbreviations: SERP1, Stress associated endoplasmic reticulum protein 1.

Supplemental Table 3. Top 50 genes most positively and negatively associated with SERP1 in SKCM patients.

Gene_name	Gene_biotype	cor_pearson	p_pearson	cor_spearman
Top 50 Positive Correlation				
SSR3	protein_coding	0.795181717	5.8818E-104	0.751330735
SLC33A1	protein_coding	0.774519189	2.45527E-95	0.725364812
MYNN	protein_coding	0.76345715	4.33617E-91	0.720671351
ZNF639	protein_coding	0.738031334	3.71758E-82	0.719719153
CNBP	protein_coding	0.752144065	5.57158E-87	0.716621838
UBA5	protein_coding	0.759276657	1.52028E-89	0.711793316
IQCB1	protein_coding	0.76088658	3.89736E-90	0.710512655
TMEM39A	protein_coding	0.748843642	7.99883E-86	0.70747931
NMD3	protein_coding	0.754461278	8.36847E-88	0.703166912
MBD4	protein_coding	0.739794515	9.65449E-83	0.701720296
EIF2A	protein_coding	0.767484819	1.31267E-92	0.696685854
ARMC8	protein_coding	0.744213481	3.13408E-84	0.69225746
ZNF148	protein_coding	0.746288972	6.11349E-85	0.68738656
DCUN1D1	protein_coding	0.742596981	1.10715E-83	0.686714244
PRKCI	protein_coding	0.742134798	1.58548E-83	0.683685263
GTF2E1	protein_coding	0.731596843	4.65073E-80	0.682707569
ZBTB11	protein_coding	0.735595434	2.35205E-81	0.682078551
TBL1XR1	protein_coding	0.740030784	8.05204E-83	0.681496161
ATR	protein_coding	0.741935038	1.85123E-83	0.680610459
NAA50	protein_coding	0.74785842	1.75761E-85	0.679028898
OSBPL11	protein_coding	0.731400519	5.37718E-80	0.674909673
SELENOT	protein_coding	0.767062889	1.89983E-92	0.674804817
PDCD10	protein_coding	0.747620414	2.12461E-85	0.673889944
DHX36	protein_coding	0.73836159	2.89033E-82	0.672835187
PIK3CA	protein_coding	0.71719975	1.40316E-75	0.670906328
RASA2	protein_coding	0.721594824	6.45089E-77	0.670582919
SENP5	protein_coding	0.721900751	5.19471E-77	0.665078521
SEC62	protein_coding	0.725632766	3.61315E-78	0.663001854
TBC1D23	protein_coding	0.722168396	4.29704E-77	0.662420267
MSL2	protein_coding	0.714728218	7.72843E-75	0.661514582
U2SURP	protein_coding	0.713729365	1.53222E-74	0.66061039
FNDC3B	protein_coding	0.701933891	3.99251E-71	0.659742375
KPNA4	protein_coding	0.72348524	1.68414E-77	0.659673352
COMMD2	protein_coding	0.754299399	9.56009E-88	0.657706134
MBNL1	protein_coding	0.72543237	4.17383E-78	0.65721275
RSRC1	protein_coding	0.723014513	2.35539E-77	0.657010848
FYTTD1	protein_coding	0.727606686	8.66561E-79	0.656022358
ATG3	protein_coding	0.720119121	1.82626E-76	0.654352824
POGLUT1	protein_coding	0.701011941	7.26358E-71	0.653684873
SKIL	protein_coding	0.679325958	5.00144E-65	0.651315345
SMC4	protein_coding	0.699104494	2.48722E-70	0.650027563
CDV3	protein_coding	0.684043148	2.96851E-66	0.649527059
NSUN3	protein_coding	0.709181273	3.33211E-73	0.649445058
MRPL3	protein_coding	0.700510064	1.00511E-70	0.648330811
XRN1	protein_coding	0.710267707	1.60547E-73	0.647459924
ZNF267	protein_coding	0.673647668	1.39746E-63	0.64645214
COPB2	protein_coding	0.719733437	2.39442E-76	0.643577054
PCNP	protein_coding	0.711978578	5.04933E-74	0.643065295
ZBTB38	protein_coding	0.690246666	6.66278E-68	0.641416778
IFT57	protein_coding	0.678044334	1.06751E-64	0.641270348
Top 50 negative correlation				
MT-CO3	protein_coding	-0.47468734	7.66882E-28	-0.535968771
MT-CO1	protein_coding	-0.467275484	6.38517E-27	-0.535862422

MT-CO2	protein_coding	-0.463413149	1.88791E-26	-0.525946284
AGPAT2	protein_coding	-0.489282133	1.01214E-29	-0.524308333
MT-ND1	protein_coding	-0.470888194	2.28736E-27	-0.496580654
MTCO1P12	unprocessed_pseudogene	-0.433029919	5.98974E-23	-0.494554749
MT-ATP6	protein_coding	-0.422009854	9.17585E-22	-0.474765195
MT-ND4	protein_coding	-0.437793391	1.78432E-23	-0.471510654
MT-ND3	protein_coding	-0.458969544	6.46072E-26	-0.470042562
MTATP6P1	unprocessed_pseudogene	-0.381584354	8.97534E-18	-0.469923351
MT-CYB	protein_coding	-0.418297813	2.24993E-21	-0.450955069
MT-ND6	protein_coding	-0.423654704	6.14458E-22	-0.443322668
GMPR	protein_coding	-0.42717949	2.5826E-22	-0.441895002
MT-ND5	protein_coding	-0.418753867	2.01638E-21	-0.437519668
C4orf48	protein_coding	-0.429169982	1.57586E-22	-0.430598515
MT-RNR1	Mt_rRNA	-0.399690235	1.7108E-19	-0.429377459
MT-ATP8	protein_coding	-0.406610286	3.5269E-20	-0.422274274
MT-RNR2	Mt_rRNA	-0.387640112	2.4526E-18	-0.41966862
TSPAN10	protein_coding	-0.417326302	2.83999E-21	-0.419311215
MT-TC	Mt_tRNA	-0.442649201	5.08996E-24	-0.413312975
MT-ND4L	protein_coding	-0.409607367	1.75939E-20	-0.410878558
MT-ND2	protein_coding	-0.390802535	1.2323E-18	-0.407848314
MT-TP	Mt_tRNA	-0.39060193	1.28758E-18	-0.402278799
MT-TY	Mt_tRNA	-0.415790896	4.09737E-21	-0.398681022
LGI3	protein_coding	-0.373791557	4.58156E-17	-0.395956283
DIPK1C	protein_coding	-0.364197867	3.21142E-16	-0.39465919
MTCO2P12	unprocessed_pseudogene	-0.330365213	1.86245E-13	-0.39454269
FNDC10	protein_coding	-0.414425212	5.66775E-21	-0.38883685
DPP7	protein_coding	-0.395165978	4.70928E-19	-0.388574195
OSGIN1	protein_coding	-0.365045958	2.71065E-16	-0.385346414
OCA2	protein_coding	-0.420111978	1.45346E-21	-0.382762991
NMRK2	protein_coding	-0.294950356	6.57745E-11	-0.37658248
NT5M	protein_coding	-0.3787931	1.61743E-17	-0.375905513
BAIAP2	protein_coding	-0.339681905	3.48552E-14	-0.371760906
REEP6	protein_coding	-0.378664848	1.66157E-17	-0.37066825
SFTPC	protein_coding	-0.274015405	1.4743E-09	-0.369830026
G6PC3	protein_coding	-0.380325905	1.17131E-17	-0.366047028
ANKRD9	protein_coding	-0.369829757	1.03199E-16	-0.362895964
TPRN	protein_coding	-0.346032998	1.07616E-14	-0.362778476
H2AJ	protein_coding	-0.333179073	1.12944E-13	-0.361549725
MTND1P23	unprocessed_pseudogene	-0.303104672	1.8251E-11	-0.361046913
DIPK1B	protein_coding	-0.369397594	1.12681E-16	-0.359836664
MRPL41	protein_coding	-0.362320507	4.66548E-16	-0.358892276
MFSD12	protein_coding	-0.350747585	4.41996E-15	-0.35865994
PMEL	protein_coding	-0.350992037	4.21893E-15	-0.35765652
MTCO1P40	processed_pseudogene	-0.269445075	2.80958E-09	-0.356970996
MTCO2P22	processed_pseudogene	-0.313498077	3.35737E-12	-0.356818702
SNTA1	protein_coding	-0.345571372	1.17319E-14	-0.352393301
SCARB1	protein_coding	-0.316987843	1.87316E-12	-0.348884948
ABCD1	protein_coding	-0.363286024	3.85134E-16	-0.347318892

Abbreviations: SERP1, Stress associated endoplasmic reticulum protein 1; SKCM, Skin Cutaneous Melanoma.

Supplementary Table 4. SERP1-interaction proteins, annotation of SERP1-interacting proteins and their co-expression scores.

Gene symbol	Annotation	Co-expression scores
SEC61B	Protein transport protein Sec61 subunit beta; Necessary for protein translocation in the endoplasmic reticulum; Belongs to the SEC61-beta family	0.983
SEC61A1	Protein transport protein Sec61 subunit alpha isoform 1; Plays a crucial role in the insertion of secretory and membrane polypeptides into the ER. Required for assembly of membrane and secretory proteins. Tightly associated with membrane- bound ribosomes, either directly or through adapter proteins. Plays a role in pronephric kidney tubule development (By similarity); Belongs to the SecY/SEC61-alpha family	0.974
SEC61G	Protein transport protein Sec61 subunit gamma; Necessary for protein translocation in the endoplasmic reticulum; Belongs to the SecE/SEC61-gamma family	0.959
ASNA1	ATPase ASNA1; ATPase required for the post-translational delivery of tail-anchored (TA) proteins to the endoplasmic reticulum. Recognizes and selectively binds the transmembrane domain of TA proteins in the cytosol. This complex then targets to the endoplasmic reticulum by membrane-bound receptors, where the tail- anchored protein is released for insertion. This process is regulated by ATP binding and hydrolysis. ATP binding drives the homodimer towards the closed dimer state, facilitating recognition of newly synthesized TA membrane proteins. ATP hydrolysis is required for insertion.	0.955
SEC62	Translocation protein SEC62; Required for preprotein translocation	0.954
SEC61A2	Protein transport protein Sec61 subunit alpha isoform 2; Appears to play a crucial role in the insertion of secretory and membrane polypeptides into the ER. It is required for assembly of membrane and secretory proteins. Found to be tightly associated with membrane-bound ribosomes, either directly or through adaptor proteins (By similarity); Belongs to the SecY/SEC61-alpha family	0.945
SEC63	Translocation protein SEC63 homolog; Required for integral membrane and secreted preprotein translocation across the endoplasmic reticulum membrane; DNAJ heat shock proteins	0.945
SPCS1	Signal peptidase complex subunit 1; Component of the microsomal signal peptidase complex which removes signal peptides from nascent proteins as they are translocated into the lumen of the endoplasmic reticulum; Belongs to the SPCS1 family	0.676
SEC11C	Signal peptidase complex catalytic subunit SEC11C; Component of the microsomal signal peptidase complex which removes signal peptides from nascent proteins as they are translocated into the lumen of the endoplasmic reticulum	0.672
DAD1	Dolichyl-diphosphooligosaccharide--protein glycosyltransferase subunit DAD1; Essential subunit of the N-oligosaccharyl transferase (OST) complex which catalyzes the transfer of a high mannose oligosaccharide from a lipid-linked oligosaccharide donor to an asparagine residue within an Asn-X-Ser/Thr consensus motif in nascent polypeptide chains. Required for the assembly of both SST3A- and SS3B-containing OST complexes. Required for efficient N-glycosylation. Loss of the DAD1 protein triggers apoptosis; Belongs to the DAD/OST2 family	0.642

Abbreviations: SERP1, Stress associated endoplasmic reticulum protein 1; SKCM, Skin Cutaneous Melanoma.

Supplementary Table 5. GO analysis and KEGG approach pathway analysis of DEGs between high and low SERP1 in SKCM patients.

Ontology	ID	Description	pvalue	p.adjust	qvalue	Count	GeneID
BP	GO:0030216	keratinocyte differentiation	2.51E-54	1.63E-51	1.49E-51	43	CDSN/DSC1/SFN/IVL/KRT1/KRT2/KRT5/KRT6A/KRT6B/KRT14/KRT16/KRT17 /PI3/SERPINB13/PKP1/S100A7/SPRR1A/SPRR1B/SPRR2A/SPRR2B/SPRR2E/ SPRR2G/FOXN1/KRT75/CASP14/KLK5/LCE2B/CNFN/LCE3D/SPRR4/KRT6C/LCE1A/LCE1B/LCE1C/ LCE1D/LCE1F/LCE2C/LCE2D/LCE3A/LCE3C/LCE3E/LCE6A/C1orf68
BP	GO:0008544	epidermis development	7.02E-53	2.28E-50	2.08E-50	47	CDSN/COL17A1/DSC1/SFN/IVL/KRT1/KRT2/KRT5/KRT6A/KRT6B/KRT14/ KRT16/KRT17/PI3/SERPINB13/PKP1/S100A7/SPRR1A/ SPRR1B/SPRR2A/SPRR2B/SPRR2E/SPRR2G/ FOXN1/KRT75/CASP14/KLK5/LCE2B/CALML5/CNFN/ LCE3D/SPRR4/KRT6C/LCE1A/LCE1B/LCE1C/ LCE1D/LCE1F/LCE2C/LCE2D/LCE3A/LCE3C/LCE3E/KRTDAP/FLG2/LCE6A/C1orf68
BP	GO:0031424	keratinization	1.09E-52	2.37E-50	2.16E-50	39	CDSN/DSC1/SFN/IVL/KRT1/KRT2/KRT5/KRT6A/KRT6B/KRT14/KRT16/KRT17/ PI3/PKP1/SPRR1A/SPRR1B/SPRR2A/SPRR2B/SPRR2E/ SPRR2G/KRT75/CASP14/KLK5/LCE2B/CNFN/ LCE3D/SPRR4/KRT6C/LCE1A/LCE1B/LCE1C/LCE1D/LCE1F/LCE2C/LCE2D/LCE3A/LCE3C/LCE3E/LCE6A
CC	GO:0001533	cornified envelope	5.83E-53	4.20E-51	3.81E-51	29	CDSN/DSC1/IVL/KRT1/KRT2/PI3/PKP1/SPRR1A/SPRR1B/SPRR2A/SPRR2B/SPRR2E/ SPRR2G/LCE2B/CNFN/LCE3D/SPRR4/LCE1A/LCE1B/LCE1C/LCE1D/LCE1F/LCE2C/ LCE2D/LCE3A/LCE3C/LCE3E/FLG2/C1orf68
CC	GO:0005882	intermediate filament	1.70E-10	6.13E-09	5.56E-09	12	KRT1/KRT2/KRT5/KRT6A/KRT6B/KRT14/KRT16/KRT17/PKP1/KRT75/CASP14/KRT6C
CC	GO:0045095	keratin filament	3.58E-10	8.59E-09	7.79E-09	9	KRT1/KRT2/KRT5/KRT6A/KRT6B/KRT14/KRT75/CASP14/KRT6C
MF	GO:0030280	structural constituent of epidermis	2.98E-13	3.67E-11	3.29E-11	7	KRT1/KRT2/PI3/PKP1/SPRR1A/SPRR2E/FLG2
MF	GO:0005200	structural constituent of cytoskeleton	3.54E-07	2.18E-05	1.96E-05	7	KRT2/KRT5/KRT6A/KRT6B/KRT14/KRT16/KRT17
MF	GO:0004867	serine-type endopeptidase inhibitor activity	6.28E-05	0.002573	0.002312	5	PI3/SERPINB13/WFDC12/A2ML1/WFDC5
KEGG	hsa04915	Estrogen signaling pathway	5.09E-06	0.000356	0.000123	5	CALML3/KRT14/KRT16/KRT17/CALML5
KEGG	hsa04912	GnRH signaling pathway	2.70E-05	0.000838	0.00029	4	CALML3/CALML5/PLA2G4E/PLA2G4F
KEGG	hsa04750	Inflammatory mediator regulation of TRP channels	3.59E-05	0.000838	0.00029	4	CALML3/CALML5/PLA2G4E/PLA2G4F

Abbreviations: SERP1, Stress associated endoplasmic reticulum protein 1; SKCM, Skin Cutaneous Melanoma; DEGs, differentially expressed genes; GO, Gene Ontology; KEGG, Kyoto Encyclopedia of Genes and Genomes; BP, biological process; MF, molecular function; CC, cellular component.

Supplementary Table 6. Top 50 enrichment plots of GSEA pathway of DEGs between high and low SERP1 in SKCM patients.

ID	pvalue	p.adjust	qvalues
REACTOME_GPCR_LIGAND_BINDING	0.001029	0.039956	0.03215
REACTOME_G_ALPHA_I_SIGNALLING_EVENTS	0.001045	0.039956	0.03215
REACTOME_CLASS_A_1_RHODOPSIN_LIKE_RECEPTORS_	0.00105	0.039956	0.03215
REACTOME_LEISHMANIA_INFECTION	0.001062	0.039956	0.03215
NABA_CORE_MATRISOME	0.001078	0.039956	0.03215
KEGG_NEUROACTIVE_LIGAND_RECEPTOR_INTERACTION	0.001079	0.039956	0.03215
KEGG_CYTOKINE_CYTOKINE_RECEPTOR_INTERACTION	0.00108	0.039956	0.03215
WP_GPCRS_CLASS_A_RHODOPSINLIKE	0.001088	0.039956	0.03215
REACTOME_ANTI_INFLAMMATORY_RESPONSE_FAVOURING_LEISHMANIA_PARASITE_INFECTION	0.001094	0.039956	0.03215
REACTOME_G_ALPHA_Q_SIGNALLING_EVENTS	0.001104	0.039956	0.03215
KEGG_CHEMOKINE_SIGNALING_PATHWAY	0.001112	0.039956	0.03215
REACTOME_PEPTIDE_LIGAND_BINDING_RECEPTORS	0.001112	0.039956	0.03215
REACTOME_CELL_SURFACE_INTERACTIONS_AT_THE_VASCULAR_WALL	0.001115	0.039956	0.03215
REACTOME_FC_EPSILON_RECEPTOR_FCERI_SIGNALING	0.001119	0.039956	0.03215
REACTOME_IMMUNOREGULATORY_INTERACTIONS_BETWEEN_A_LYMPHOID_AND_A_NON_LYMPHOID_CELL	0.001119	0.039956	0.03215
REACTOME_FCGAMMA_RECEPTOR_FCGR_DEPENDENT_PHAGOCYTOSIS	0.001153	0.039956	0.03215
REACTOME_SIGNALING_BY_THE_B_CELL_RECEPTOR_BCR_	0.001153	0.039956	0.03215
WP_CHEMOKINE_SIGNALING_PATHWAY	0.001156	0.039956	0.03215
KEGG_JAK_STAT_SIGNALING_PATHWAY	0.001157	0.039956	0.03215
REACTOME_FCERI_MEDIATED_NF_KB_ACTIVATION	0.001164	0.039956	0.03215
KEGG_CELL_ADHESION_MOLECULES_CAMS	0.001167	0.039956	0.03215
REACTOME_COMPLEMENT_CASCADE	0.001192	0.039956	0.03215
REACTOME_PARASITE_INFECTION	0.001198	0.039956	0.03215
REACTOME_BINDING_AND_UPTAKE_OF_LIGANDS_BY_SCAVENGER_RECEPTORS	0.001229	0.039956	0.03215
WP_HUMAN_COMPLEMENT_SYSTEM	0.00123	0.039956	0.03215
REACTOME_FCERI_MEDIATED_MAPK_ACTIVATION	0.001232	0.039956	0.03215
REACTOME_FCGR3A_MEDIATED_IL10_SYNTHESIS	0.001236	0.039956	0.03215
REACTOME_ANTIGEN_ACTIVATES_B_CELL_RECEPTOR_BCR_LEADING_TO_GENERATION_OF_SECOND_MESSENGERS	0.001239	0.039956	0.03215
REACTOME_FCERI_MEDIATED_CA_2_MOBILIZATION	0.001239	0.039956	0.03215
REACTOME_INITIAL_TRIGGERING_OF_COMPLEMENT	0.001241	0.039956	0.03215
WP_ALLOGRAFT_REJECTION	0.001241	0.039956	0.03215
REACTOME_ROLE_OF_PHOSPHOLIPIDS_IN_PHAGOCYTOSIS	0.001247	0.039956	0.03215
WP_TCELL_ANTIGEN_RECEPTOR_TCR_SIGNALING_PATHWAY	0.001248	0.039956	0.03215
KEGG_HEMATOPOIETIC_CELL_LINEAGE	0.001255	0.039956	0.03215
WP_PEPTIDE_GPCRS	0.001255	0.039956	0.03215
KEGG_LEISHMANIA_INFECTION	0.001272	0.039956	0.03215
REACTOME_CREATION_OF_C4_AND_C2_ACTIVATORS	0.001277	0.039956	0.03215
REACTOME_ROLE_OF_LAT2_NTAL_LAB_ON_CALCIUM_MOBILIZATION	0.001277	0.039956	0.03215
REACTOME_FCGR_ACTIVATION	0.001284	0.039956	0.03215
REACTOME_SCAVENGING_OF_HEME_FROM_PLASMA	0.001284	0.039956	0.03215
PID_IL12_2PATHWAY	0.001321	0.039956	0.03215
WP_TCELL_ANTIGEN_RECEPTOR_TCR_PATHWAY_DURING_STAPHYLOCOCCUS_AUREUS_INFECTION	0.001321	0.039956	0.03215
REACTOME_CD22_MEDIATED_BCR_REGULATION	0.001325	0.039956	0.03215
PID_TCR_PATHWAY	0.001328	0.039956	0.03215
PID_CD8_TCR_PATHWAY	0.001346	0.039956	0.03215
REACTOME_INTERLEUKIN_10_SIGNALING	0.001346	0.039956	0.03215
KEGG_INTESTINAL_IMMUNE_NETWORK_FOR_IGA_PRODUCTION	0.001353	0.039956	0.03215
REACTOME_CHEMOKINE_RECEPTORS_BIND_CHEMOKINES	0.001353	0.039956	0.03215
REACTOME_INTERLEUKIN_2_FAMILY_SIGNALING	0.001366	0.039956	0.03215
PID_IL12_STAT4_PATHWAY	0.001403	0.039956	0.03215

Abbreviations: SERP1, Stress associated endoplasmic reticulum protein 1; SKCM, Skin Cutaneous Melanoma; DEGs, differentially expressed genes; GSEA, Gene set enrichment analyses.

Supplementary Table 7. Univariate and multivariate Cox regression analysis for clinical variables associated with OS in SKCM patients.

Characteristics	Total(N)	Univariate analysis		Multivariate analysis	
		Hazard ratio (95% CI)	P value	Hazard ratio (95% CI)	P value
SERP1 (High vs. Low)	456	0.625 (0.476-0.820)	**<0.001**	0.591 (0.405-0.861)	**0.006**
T stage (T3&T4&T2 vs. T1)	361	2.255 (1.303-3.903)	**0.004**		
N stage (N1&N2&N3 vs. N0)	402	1.752 (1.304-2.354)	**<0.001**	2.945 (1.887-4.597)	**<0.001**
M stage (M1 vs. M0)	430	1.897 (1.029-3.496)	**0.040**		
Gender (Male vs. Female)	456	1.172 (0.879-1.563)	0.281		
Age (>60 vs. <=60)	456	1.656 (1.251-2.192)	**<0.001**	1.204 (0.828-1.750)	0.331
Race (White vs. Asian&Black or African American)	446	0.226 (0.104-0.489)	**<0.001**	0.445 (0.170-1.160)	0.098
Radiation therapy (Yes vs. No)	450	0.977 (0.694-1.377)	0.895		
Melanoma ulceration (Yes vs. No)	313	2.085 (1.495-2.907)	**<0.001**	1.645 (1.062-2.546)	**0.026**
Breslow depth (>3 vs. <=3)	355	2.651 (1.938-3.627)	**<0.001**	2.028 (1.312-3.136)	**0.001**
Pathologic stage (Stage II&Stage III&Stage IV vs. Stage I)	410	1.846 (1.292-2.638)	**<0.001**	0.563 (0.303-1.046)	0.069
BMI (>25 vs. <=25)	241	0.827 (0.513-1.333)	0.436		
Tumor tissue site (Extremities&Head and Neck&Other Specify vs. Trunk)	405	1.147 (0.859-1.532)	0.353		
Melanoma Clark level (III&IV&V vs. I&II)	315	2.689 (1.188-6.088)	**0.018**		

Abbreviations: SERP1, Stress associated endoplasmic reticulum protein 1; SKCM, Skin Cutaneous Melanoma; OS, overall survival; CI, confidence interval. Bold values indicate $P < 0.05$.

Supplementary Table 8. Univariate and multivariate Cox regression analysis for clinical variables associated with DSS in SKCM patients.

Characteristics	Total(N)	Univariate analysis		Multivariate analysis	
		Hazard ratio (95% CI)	P value	Hazard ratio (95% CI)	P value
T stage (T3&T4&T2 vs. T1)	356	2.026 (1.166-3.518)	**0.012**		
N stage (N1&N2&N3 vs. N0)	396	1.665 (1.214-2.283)	**0.002**	2.893 (1.813-4.616)	**<0.001**
M stage (M1 vs. M0)	424	2.200 (1.190-4.069)	**0.012**		
Gender (Male vs. Female)	450	1.161 (0.855-1.575)	0.340		
Age (>60 vs. <=60)	450	1.699 (1.258-2.294)	**<0.001**	1.184 (0.799-1.756)	0.400
Race (White vs. Asian&Black or African American)	440	0.464 (0.146-1.474)	0.193		
Radiation therapy (Yes vs. No)	444	0.994 (0.689-1.433)	0.973		
Melanoma ulceration (Yes vs. No)	309	1.948 (1.372-2.767)	**<0.001**	1.709 (1.087-2.687)	**0.020**
Breslow depth (>3 vs. <=3)	350	2.274 (1.628-3.177)	**<0.001**	1.719 (1.096-2.695)	**0.018**
SERP1 (High vs. Low)	450	0.655 (0.490-0.874)	**0.004**	0.584 (0.395-0.864)	**0.007**
Pathologic stage (Stage II&Stage III&Stage IV vs. Stage I)	405	1.711 (1.181-2.478)	**0.004**	0.586 (0.309-1.109)	0.101
Tumor tissue site (Extremities&Head and Neck&Other Specify vs. Trunk)	399	1.183 (0.868-1.611)	0.288		
BMI (>25 vs. <=25)	238	0.937 (0.545-1.612)	0.815		
Melanoma Clark level (III&IV&V vs. I&II)	310	2.949 (1.207-7.209)	**0.018**		

Abbreviations: SERP1, Stress associated endoplasmic reticulum protein 1; SKCM, Skin Cutaneous Melanoma; DSS, disease specific survival; CI, confidence interval. Bold values indicate P<0.05.

Supplementary Table 9. Univariate and multivariate Cox regression analysis for clinical variables associated with PFI in SKCM patients.

Characteristics	Total(N)	Univariate analysis		Multivariate analysis	
		Hazard ratio (95% CI)	P value	Hazard ratio (95% CI)	P value
T stage (T3&T4 vs. T1&T2)	362	1.664 (1.268-2.183)	**<0.001**	1.489 (0.795-2.789)	0.214
N stage (N1&N2&N3 vs. N0)	403	1.870 (1.467-2.385)	**<0.001**	2.925 (1.975-4.334)	**<0.001**
M stage (M1 vs. M0)	431	2.026 (1.255-3.269)	**0.004**	1.519 (0.686-3.364)	0.302
Pathologic stage (Stage II&Stage III&Stage IV vs. Stage I)	411	1.739 (1.293-2.340)	**<0.001**	0.405 (0.195-0.839)	**0.015**
Radiation therapy (Yes vs. No)	451	1.214 (0.917-1.606)	0.175		
Gender (Male vs. Female)	457	1.037 (0.821-1.309)	0.763		
Race (White vs. Asian&Black or African American)	447	0.965 (0.358-2.605)	0.945		
Age (>60 vs. <=60)	457	1.576 (1.240-2.002)	**<0.001**	1.362 (0.971-1.911)	0.074
Melanoma ulceration (Yes vs. No)	313	1.626 (1.228-2.152)	**<0.001**	1.536 (1.040-2.267)	**0.031**
Melanoma Clark level (III&IV&V vs. I&II)	315	1.864 (1.039-3.346)	**0.037**	1.122 (0.551-2.288)	0.751
BMI (>25 vs. <=25)	241	0.966 (0.660-1.416)	0.861		
Breslow depth (>3 vs. <=3)	355	2.032 (1.547-2.669)	**<0.001**	1.557 (1.003-2.417)	**0.048**
SERP1 (High vs. Low)	457	0.768 (0.612-0.964)	**0.023**	0.769 (0.555-1.067)	0.116

Abbreviations: SERP1, Stress associated endoplasmic reticulum protein 1; SKCM, Skin Cutaneous Melanoma; PFI, progress free interval; CI, confidence interval. Bold values indicate P<0.05.

Supplementary Table 10. Details of the ROC information.

Time	Cut-off	Sensitivity	Specificity	Positive predictive value	Negative predictive value
OS					
1 year	6.255538	0.179	0.628	0.0322	0.917
3 year	5.896933	0.516	0.295	0.231	0.598
5 year	6.190405	0.302	0.523	0.308	0.515
DSS					
1 year	6.500816	0.0459	0.789	0.0117	0.938
3 year	5.903546	0.527	0.299	0.208	0.644
5 year	6.190405	0.308	0.52	0.287	0.545

Abbreviations: overall survival: OS; disease specific survival: DSS
